# Senataxin regulates cisplatin resistance through an R-loop-mediated mechanism in HPV-associated head and neck cancer

**DOI:** 10.1016/j.isci.2025.113348

**Published:** 2025-08-13

**Authors:** Hannah Crane, Ian Carr, Keith D. Hunter, Sherif F. El-Khamisy

**Affiliations:** 1 School of Clinical Dentistry, University of Sheffield, Sheffield, UK; 2 The Healthy Lifespan Institute, University of Sheffield, Sheffield, UK; 3 School of Biosciences, University of Sheffield, Sheffield, UK; 4 Leeds Institute of Medical Research, University of Leeds, Leeds, UK; 5 Liverpool Head and Neck Centre, Molecular and Clinical Cancer Medicine, University of Liverpool, Liverpool, UK; 6 Institute of Cancer Therapeutics, University of Bradford, Bradford, UK

**Keywords:** cancer, genomic analysis, genomics

## Abstract

Resistance to cisplatin is a key clinical concern in HPV-independent (HPV−) and HPV-associated (HPV+) head and neck cancer. Upregulation of DNA repair is known to contribute to cisplatin resistance and a major source of endogenous DNA damage are DNA/RNA hybrids, known as R-loops. Following creation of HPV+ and HPV− cisplatin resistant cell lines, RNA-sequencing revealed alterations in the expression of known R-loop regulators. Resistant cells had elevated global R-loop levels and in HPV+ resistant cells there was a corresponding upregulation of the R-loop resolving protein, senataxin. Depletion of senataxin led to increased sensitivity to cisplatin, an increase in DNA damage and elevated R-loops at specific genomic loci. In summary, using an *in vitro* model of cisplatin resistance, we identified that senataxin modulates sensitivity to cisplatin through an R-loop-mediated mechanism in HPV+ cells. R-loops may represent a potential therapeutic target and warrant further investigation.

## Highlights

• Cisplatin resistant cells show differential expression of R-loop regulators
• Global R-loops were elevated in resistant cells
• Senataxin depletion led to increased sensitivity to cisplatin
• Senataxin depletion was associated with increased DNA damage and elevated R-loops

## Graphical abstract

**Figure:**
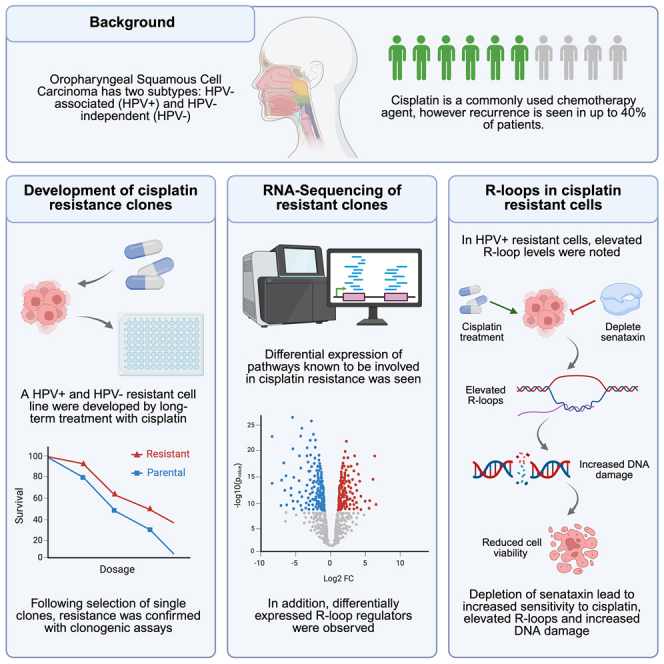

## Introduction

Oropharyngeal squamous cell carcinoma (OPSCC) is a site defined subset of head and neck squamous cell carcinoma occurring in the base of tongue, soft palate, and tonsils,^1^ which has shown a dramatic increase in incidence in the western world.^2^ Over the past three decades, the role of high risk human papilloma virus (HPV), in a subset of OPSCC has become apparent.^3^ Given the overwhelming evidence of a causative role of HPV, in 2017 OPSCC was categorized into two distinct clinical subtypes, HPV-positive (HPV-associated/HPV+) and HPV-negative (HPV-independent/HPV−).^1^^,^^4^ This was undertaken in recognition of the markedly improved prognosis of HPV+ OPSCC (3-year survival of 82.4%) when compared to HPV− OPSCC (3-year survival of 57.1%).^3^

Platinum-based chemotherapeutic agents, such as cisplatin are a mainstay in the treatment of OPSCC, alongside surgery and radiotherapy. Cisplatin (*cis*-diamminedichloroplatinum [II]) was first identified as an anti-tumor agent in 1969^5^ and following cisplatin uptake into a tumor cell, the low chloride concentration within the cytoplasm allows for replacement of chloride ions with water.^6^ This “aquated” cisplatin is able to bind to nuclear and mitochondrial DNA with high affinity, causing intra- and inter-strand DNA crosslinks, blocking transcription pathways and leading to eventual cell death by apoptosis.^7^

A systematic review has shown that chemotherapy, when used alongside surgery and radiotherapy, is associated with improved survival in patients with oral and oropharyngeal cancer.^8^ Furthermore, in HPV+ OPSCC, a trial of treatment de-escalation through replacement of cisplatin with the monoclonal antibody cetuximab resulted in inferior clinical outcomes.^9^ Therefore, platinum based chemotherapies, such as cisplatin, continue to be an important component of treatment for patients with HPV+ and HPV− OPSCC, improving clinical outcomes.

Notwithstanding the importance of cisplatin in the treatment of OPSCC, resistance to treatment is common, especially in HPV− OPSCC which persists with poor overall survival.^3^ Despite the overall improved prognosis of HPV+ OPSCC, there are a subset of patients who present with loco-regional recurrences or distant metastases, with a poor prognosis.^10^^,^^11^^,^^12^^,^^13^ A systematic review also highlighted that patients with HPV+ OPSCC were more likely to undergo distant metastases to multiple organs when compared to HPV− OPSCC.^14^ Therefore, it is imperative to understand the molecular basis of cisplatin resistance in both HPV+ and HPV− OPSCC.

Resistance to cisplatin is multifactorial and researchers have demonstrated the importance of a number of factors including tumor heterogeneity, altered cellular uptake and effect of the surrounding tumor microenvironment.^15^ The DNA damage response is also known to be crucial in mediating resistance to cisplatin therapies.^16^ A major source of endogenous DNA damage are DNA/RNA hybrids (R-loops). R-loops were first described in 1979 and are three stranded nucleic structures comprised of a DNA:RNA hybrid with an associated free strand of DNA.^17^ R-loops form as a by-product of transcription and next-generation sequencing studies have shown they occupy 5%–10% of the genome,^18^^,^^19^ preferentially forming in regions of the genome which are G-rich or show GC-skew.^20^^,^^21^ R-loops are known to have a number of physiological roles, including enabling transcriptional activation through prevention of DNA methylation at promoter regions.^22^ However, unscheduled or persistent R-loops are a potential source of genomic instability, largely due to their potential to cause transcription-replication conflicts.^23^ R-loops have been implicated in the pathogenesis of a number of tumors, including Embryonal Tumors with Multilayered Rosettes (ETMR)^24^ and Ewing sarcoma.^25^ However, the contribution of R-loops to the development of cisplatin resistance in OPSCC has not previously been investigated.

We hypothesized that R-loop physiology would change upon the development of cisplatin resistance and that it could be modulated for therapeutic benefit. To investigate this, we developed cisplatin resistant clones of an HPV+ and HPV− cell line and utilized these to explore R-loop dynamics upon the development of resistance.

## Results

### Long term treatment with cisplatin results in resistant clones which show differential expression of R-loop regulators

**Figure 1:**
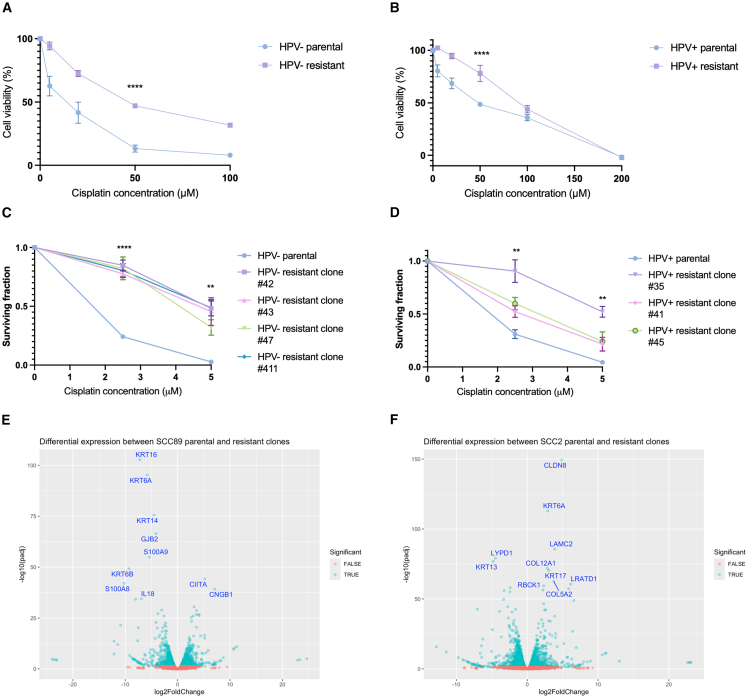
Long-term treatment with cisplatin results in the development of resistant clones and RNA-sequencing reveals differential expression of genes and pathways known to be involved in cisplatin resistance (A) Cell viability assessed with an MTS assay following long-term treatment with cisplatin in HPV− (SCC89) parental cells (IC50: 10.0 μM) and resistant cells (IC50: 47.6 μM; *n* = 3, mean ± sem). (B) Cell viability assessed with an MTS assay following treatment with long term cisplatin in HPV+ (SCC2) parental cells (IC50: 40.0 μM) and resistant cells (IC50: 84.9 μM; *n* = 3, mean ± sem). (C) Results of clonogenic assay quantification to assess differences in surviving fraction of HPV− parental cells and resistant clones to cisplatin treatment (*n* = 3, mean ± sem). (D) Results of clonogenic assay quantification to assess differences in surviving fraction of HPV+ parental cells and resistant clones to cisplatin treatment (*n* = 3, mean ± sem). (E) Volcano plot demonstrating differentially expressed genes in the HPV− resistant clone compared to parental cells, with the top 10 differentially expressed genes labeled. (F) Volcano plot demonstrating differentially expressed genes in the HPV+ resistant clone compared to parental cells, with the top 10 differentially expressed genes labeled. Statistical analysis carried out in (A) and (B) using non-linear regression and extra sum-of-squares F test was used to compare LogIC50 between datasets. Statistical analysis carried out in C and D using one way-ANOVA at both 2.5 and 5 μM concentrations with Tukey’s post-hoc test. Differential expression conducted in E and F using DESeq2 with differentially expressed genes meeting the criteria of an adjusted *p* value less than 0.05 and a log2 fold change of greater than 1. ^a^ns = not significant, ∗*p* < 0.05, ∗∗*p* ≤ 0.01, ∗∗∗*p* ≤ 0.001, ∗∗∗∗*p* ≤ 0.0001.

In order to investigate platinum resistance, cisplatin resistant cells were developed using long term cisplatin treatment of an HPV+ and HPV− cell line, which led to a statistically significant increase in the half maximal inhibitory concentration (IC50) in both cell lines (Figures 1A and 1B). HPV− resistant cells showed an increase in IC50 from 10.0 to 47.6 μM (*p* < 0.0001), whereas in the HPV+ resistant cells the IC50 increased from 40.0 to 84.9 μM (*p* < 0.0001). Following selection of single cell clones, clonogenic assays were performed to confirm the resistance was stable and established that the selected clones were more resistant to cisplatin treatment compared to the parental cells (Figures 1C and 1D).

**Figure 2:**
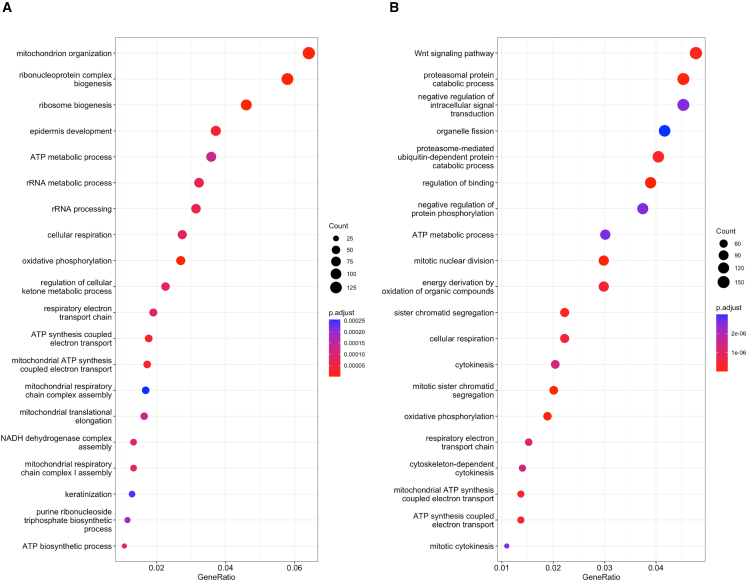
Pathway enrichment analysis in HPV− and HPV+ resistant cells (A) Gene expression analysis of differentially expressed genes in HPV− resistant cells compared to parental cells using clusterProlifer. (B) Gene expression analysis of differentially expressed genes in HPV+ resistant cells compared to parental cells using clusterProlifer. The genes investigated within the pathway enrichment analysis were those resulting from the DESeq2 analysis, with genes meeting the criteria of an adjusted *p* value less than 0.05 and a log2 fold change of greater than 1.

A selected resistant clone and associated parental cells were subjected to RNA-sequencing to explore transcriptome wide changes and confirm the clones were an appropriate model of cisplatin resistance. Upon development of resistance, there were 1,521 and 1,234 differentially expressed transcripts in the HPV− and HPV+ cells respectively (Figures 1E and 1F). Using clusterProlifer, gene pathway analysis of the significantly differentially expressed genes (as highlighted on the volcano plots) revealed a number of differentially expressed pathways (Figures 2A and 2B). The Wnt signaling pathway was the most over-represented pathway upon development of cisplatin resistance in HPV+ cells (Figure 2B), and this pathway has previously been associated with cisplatin chemoresistance in lung adenocarcinoma.^26^ Interestingly, the oxidative phosphorylation pathway was over-represented in both HPV+ and HPV− resistant cells (Figures 2A and 2B). Cisplatin treatment is known to increase levels of metabolites associated with generalized oxidative stress,^27^^,^^28^^,^^29^ and previous studies have demonstrated that cisplatin resistance results in differential gene expression with an enrichment in genes associated with oxidative phosphorylation.^27^ Furthermore, oxidative phosphorylation has also been associated with resistance to other chemotherapeutic agents including MEK inhibitors clinically utilized in KRAS mutant lung cancer^30^ and metastatic endocrine resistant breast cancer.^31^

**Figure 3:**
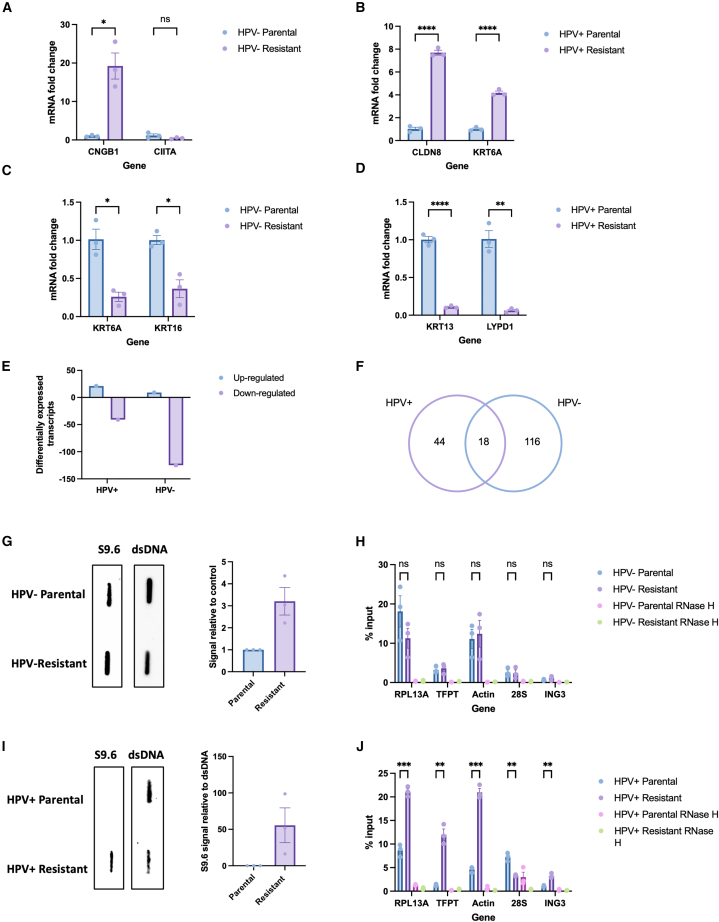
Upon development of resistance, HPV+ and HPV− cells show alterations in R-loop burden (A–D) Validation of two upregulated (A) and two downregulated (C) transcripts in the HPV− resistant cells using RT-qPCR (*n* = 3, mean ± sem). Validation of two upregulated (B) and two downregulated (D) transcripts in the HPV+ resistant cells using RT-qPCR (*n* = 3, mean ± sem). (E) Number of differentially expressed R-loop regulators in the HPV+ and HPV− resistant cells. (F) Venn diagram to illustrate the overlap in differentially expressed R-loop regulators. (G) S9.6 slot blot to evaluate global differences in R-loops at baseline between HPV− parental and resistant cells, with quantification of three repeats (mean ± sem). (H) Percentage input at positive R-loop loci in HPV− parental and resistant cells in untreated conditions, with associated RNase H treated controls (*n* = 3, mean ± sem). (I) S9.6 slot blot to evaluate global differences in R-loops at baseline between HPV+ parental and resistant cells, with quantification of three repeats (mean ± sem). dsDNA (double-stranded DNA) was used as a loading control in (G) and (I). (J) Percentage input at positive R-loop loci in HPV+ parental and resistant cells in untreated conditions, with associated RNase H treated controls (*n* = 3, mean ± sem). Statistics carried out in (A–D), (H), and (I) using multiple unpaired t tests (*n* = 3, mean ± sem). ns = not significant, ∗*p* < 0.05, ∗∗*p* ≤ 0.01, ∗∗∗*p* ≤ 0.001, ∗∗∗∗*p* ≤ 0.0001. For all figures, where indicated DNA was treated with RNase H sourced from NEB (M0297).

From the top ten differentially expressed genes in Figures 1E and 1F, two upregulated and two downregulated genes were validated with qPCR for both the HPV+ and HPV− clones (Figures 3A–3D), and successful validation was achieved for 7 of these targets. Differential expression of genes known to be implicated in development of cisplatin resistance in other tumor types were identified, including upregulation of cyclic nucleotide gated channel subunit Beta 1 (CNGB1) in HPV− resistant cells (Figure 3A). CNGB1 controls intracellular cation levels and is associated with cisplatin resistance and reduced progression free survival in bladder cancer.^32^ Upon development of resistance in the HPV+ cells, overexpression of keratin 6A (KRT6A) was observed (Figure 3B). KRT6A is an intermediate filament providing structure to epithelial cells and overexpression of KRT6A has previously been reported in a cisplatin resistant variant of a gastric adenocarcinoma cell line.^33^ Interestingly, although keratinization was a significantly altered pathway in the HPV− cells (Figure 2A), KRT6A was downregulated in the HPV− resistant cells (Figure 3C), suggesting HPV+ and HPV− cells have differing gene expression profiles upon development of cisplatin resistance. Taken together, these findings indicate that the clones chosen are an appropriate model of cisplatin resistance.

Given that oxidative phosphorylation pathways were differentially expressed in resistant cells and reactive oxygen species (ROS) have previously been shown to increase R-loop levels,^34^^,^^35^^,^^36^ we next explored if there was differential expression of known R-loop regulators, using the publicly available database R-loopBase.^37^ R-loopBase is a database which utilizes multi-omics analysis and literature searching to collate tiers of known R-loop regulators, with the highest tier of regulator having been validated in multiple *in vitro* assays.^37^ Using this database there were 134 and 62 differentially expressed R-loop regulators in the HPV− and HPV+ resistant cells, respectively (Figures 3E and 3F).

### Cisplatin resistant HPV+ cells have an increase in global R-loops, with an associated upregulation of senataxin expression

Having confirmed the clones were an appropriate model of cisplatin resistance and following identification of differential expression of R-loop regulators, we next sought to explore changes in global R-loop levels using an S9.6 slot blot. This revealed a global increase in R-loops in both HPV+ and HPV− cells upon the development of cisplatin resistance, with a larger increase noticed in the HPV+ cells (Figures 3G and 3I). However, when R-loop burden was investigated at specific genomic R-loop loci using DRIP-qPCR (*RPL13A*, *TFPT*, *Actin*, *28S* and *ING3*^38^^,^^39^^,^^40^), we found an increase in R-loop occupancy in the HPV+ resistant cells (Figure 3J), which was not identified in the HPV− resistant cells (Figure 3H). This may be explained by alterations at loci other than those investigated with qPCR or that the magnitude of the global increase was larger in the HPV+ cells leading to associated increases at specific loci which were detectable by qPCR. Treatment with 24 h of cisplatin led to an increase in global R-loop levels in both HPV+ and HPV− cells (Figures S2A–S2D). A comparable increase in global R-loop level was identified following cisplatin treatment in HPV+ resistant cells, however, in HPV− resistant cells there was no observed increase following cisplatin treatment (Figures S2A–S2D).

**Figure 4:**
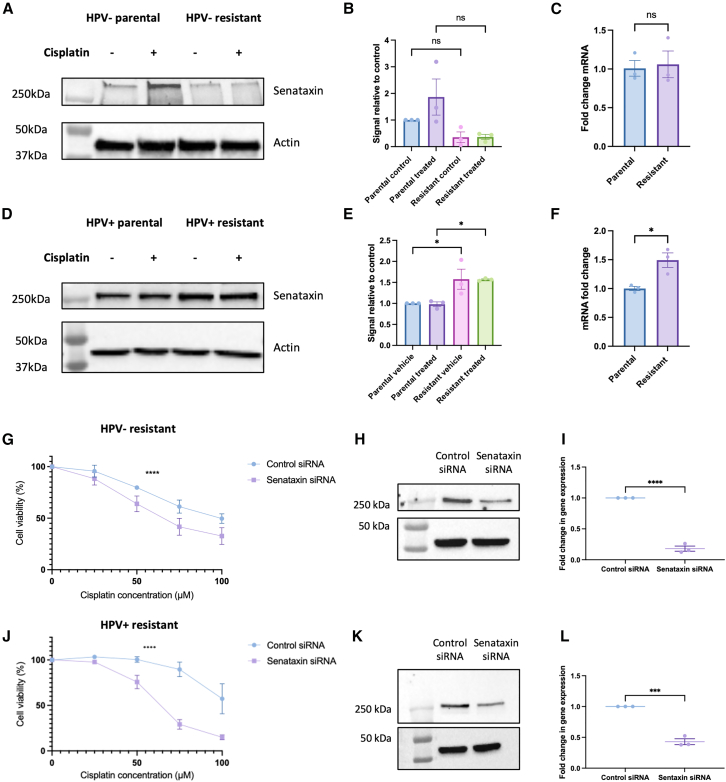
HPV+ resistant cells upregulate senataxin upon development of cisplatin resistance and showed reduced cell viability following cisplatin treatment in the presence of senataxin knockdown (A) Western blot of senataxin expression in HPV− parental and resistant cells, following 5 μM cisplatin treatment for 24 h compared to vehicle only control. (B) Quantification of three repeats of A (*n* = 3, mean ± sem). (C) Senataxin mRNA expression in untreated HPV− parental and resistant cells (*n* = 3, mean ± sem). (D) Western blot of senataxin expression in HPV+ parental and resistant cells, following 5 μM cisplatin treatment for 24 h compared to vehicle only control. (E) Quantification of three repeats of D (*n* = 3, mean ± sem). (F) Senataxin mRNA expression in untreated HPV+ parental and resistant cells (*n* = 3, mean ± sem). (G) Cell viability in HPV− resistant cells in response to cisplatin in presence of senataxin knockdown compared to control (scrambled) siRNA (*n* = 3, mean ± sem). IC50 for control siRNA: 96.93 μM, IC50 for senataxin siRNA: 66.37 μM. (H) Confirmation of knockdown in HPV− resistant cells with western blot. (I) Confirmation of knockdown in HPV− resistant cells using qPCR (*n* = 3, mean ± sem). (J) Cell viability in HPV+ resistant cells in response to cisplatin in presence of senataxin knockdown compared to control (scrambled) siRNA (*n* = 3, mean ± sem). IC50 for control siRNA: 104.6 μM, IC50 for senataxin siRNA: 63.41 μM. (K) Confirmation of knockdown in HPV+ resistant cells with western blot. (L) Confirmation of knockdown in HPV+ resistant cells using qPCR (*n* = 3, mean ± sem). Statistical analysis carried out in B and E using one way-ANOVA. Statistical analysis carried on C, F, I, and L using unpaired t test (with Welch’s correction in I and L). Statistics carried out in G and J using non-linear regression and extra sum-of-squares F test to compare LogIC50 between datasets. ns = not significant, ∗*p* < 0.05, ∗∗*p* ≤ 0.01, ∗∗∗*p* ≤ 0.001, ∗∗∗∗*p* ≤ 0.0001.

Senataxin is an helicase which is known to resolve R-loops at transcription termination sites^38^ and has recently been shown to be important in mediating R-loop resolution on HPV episomes to allow for transcription of viral oncoproteins.^41^ Senataxin modulation has previously been successfully used as a model of R-loop biology^42^ and a recent genome wide CRISPR screen highlighted that senataxin knockout sensitized retinal pigment epithelium-1 (RPE1) cells to cisplatin induced DNA damage.^43^ Therefore, we initially explored if there were any alterations in senataxin expression upon development of resistance. There was no statistically significant increase in senataxin protein or mRNA levels in the HPV− resistant cells (Figures 4A–4C), however, there was a small but statistically significant upregulation of senataxin protein upon development of cisplatin resistance in HPV+ cells (Figures 4D and 4E), which was mirrored by an increase in senataxin mRNA levels (Figure 4F).

### Both HPV+ and HPV− resistant cells are sensitized to cisplatin upon senataxin depletion

In order to model the effect of increasing R-loops in the context of cisplatin resistance, we next investigated if reduction of senataxin protein levels could modulate the response to cisplatin in resistant cells. Senataxin siRNA led to a successful reduction in protein and mRNA levels in HPV− resistant cells (Figures 4H and 4I) and HPV+ resistant cells (Figures 4K and 4L). In the HPV+ resistant cells, depletion of senataxin led to a marked reduction in cell viability in response to cisplatin (Figure 4J). Depletion of senataxin in the HPV− resistant cells also led to a reduction in cell viability following treatment with cisplatin, however, the effect was smaller compared to the HPV+ resistant cells (Figure 4G).

### Depletion of senataxin leads to elevated DNA double-strand breaks and R-loops following cisplatin treatment

**Figure 5:**
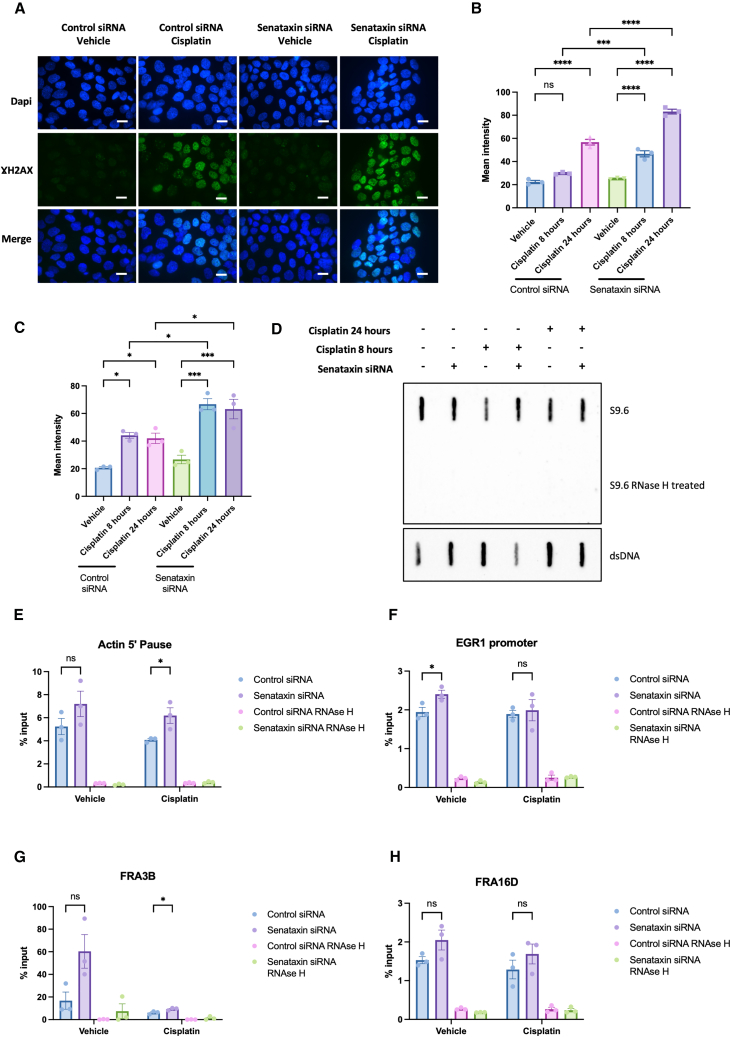
Knockdown of senataxin results in increased DNA damage and R-loops following cisplatin treatment (A) Representative images of Gamma H2AX immunofluorescence after 24 h of 25 μM cisplatin treatment compared to vehicle only control in HPV+ resistant cells. Scale bars, 10 μm. (B) Quantification of mean nuclear intensity following senataxin knockdown and 25 μM cisplatin treatment in HPV+ resistant cells for 8 or 24 h as indicated (*n* = 3, mean ± sem). (C) Quantification of mean nuclear intensity following senataxin knockdown and 25 μM cisplatin treatment in HPV− resistant cells for 8 or 24 h as indicated (*n* = 3, mean ± sem). (D) S9.6 slot blot to investigate global changes in R-loops following senataxin knockdown and 25 μM cisplatin treatment for 8 and 24 h in HPV+ resistant cells. (E) DRIP-qPCR in HPV+ resistant cells following senataxin knockdown and vehicle or cisplatin treatment at Actin 5′ pause locus (*n* = 3, mean ± sem). (F) DRIP-qPCR in HPV+ resistant cells following senataxin knockdown and vehicle or cisplatin treatment at EGR1 promoter locus (*n* = 3, mean ± sem). (G) DRIP-qPCR in HPV+ resistant cells following senataxin knockdown and vehicle or cisplatin treatment at FRA3B locus (*n* = 3, mean ± sem). (H) DRIP-qPCR in HPV+ resistant cells following senataxin knockdown and vehicle or cisplatin treatment at FRA16D locus (*n* = 3, mean ± sem). For quantification of immunofluorescence images, at least 50 cells were quantified per biological repeat. Statistical analysis carried out on B and C with one-way ANOVA and multiple comparisons carried out using post-hoc Tukey’s test. Statistics carried out on E–H using unpaired t tests. ns = not significant, ∗*p* < 0.05, ∗∗*p* ≤ 0.01, ∗∗∗*p* ≤ 0.001, ∗∗∗∗*p* ≤ 0.0001.

After identifying that depletion of senataxin affected sensitivity to cisplatin in both HPV+ and HPV− resistant cells, we next went onto explore the effect of senataxin depletion on DNA damage using γH2AX immunofluorescence. In both HPV+ and HPV− resistant cells, there was an increase in DNA damage following treatment with cisplatin, however, the increase in the senataxin depleted cells was significantly greater (Figures 5A–5C). Successful knockdown was confirmed using senataxin immunofluorescence (Figures S4A–S4D). With γH2AX immunofluorescence, there was no increase in DNA damage in the presence of senataxin knockdown alone. These findings were confirmed with an alkaline comet assay under the same conditions in both HPV+ and HPV− resistant cells (Figures S3A–S3B). Following these observations, we next examined whether the reduced cell viability and associated increase in DNA damage noted in the presence of senataxin knockdown was mediated through alterations in R-loop levels. As seen in Figures 5E–5H, following cisplatin treatment in senataxin depleted HPV+ resistant cells, there was an increase in R-loop occupancy at certain genomic loci, such as actin 5′ pause. This correlated with a global increase in R-loops under the same conditions (Figure 5D).

### USP11 re-sensitizes HPV− resistant cells to cisplatin

**Figure 6:**
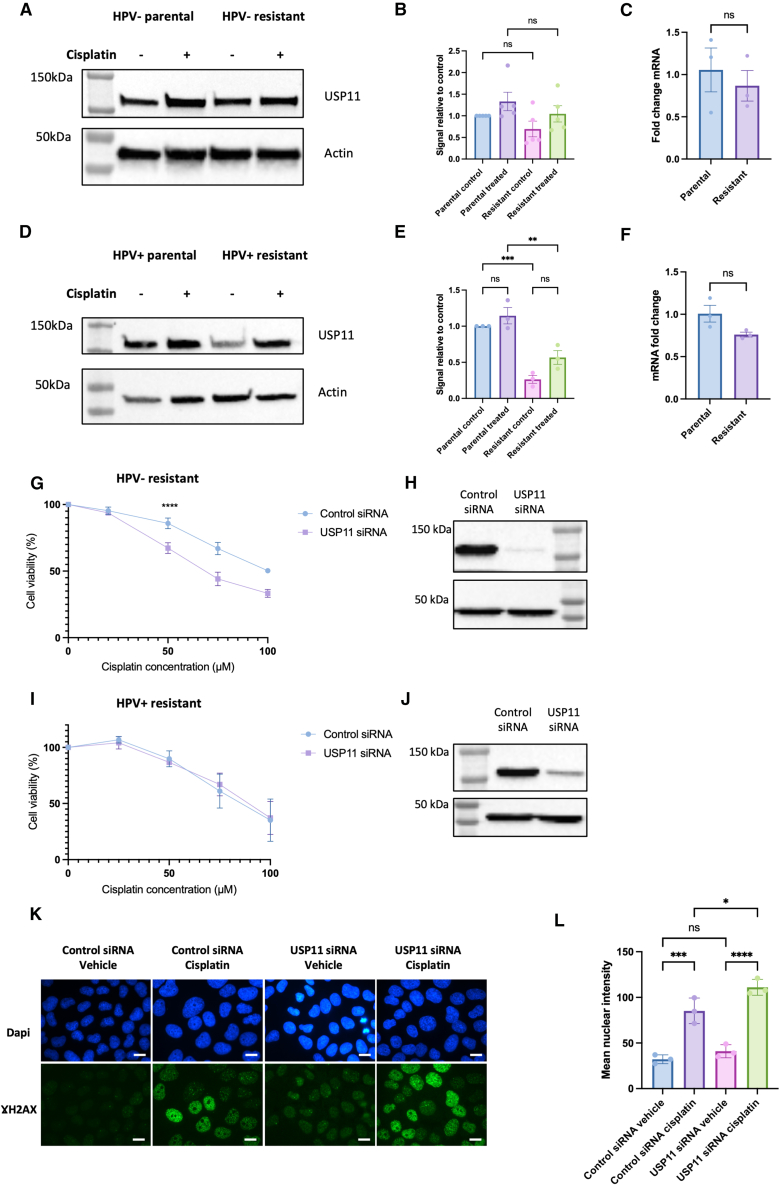
USP11 re-sensitizes HPV− resistant cells to cisplatin (A) Western blot of USP11 expression in HPV− parental and resistant cells, following 5 μM cisplatin treatment for 24 h compared to vehicle only control. Actin control is also present in Figure 4A, which was part of the same experiment. (B) Quantification of three repeats of A (*n* = 3, mean ± sem). (C) USP11 mRNA expression in untreated HPV− parental and resistant cells (*n* = 3, mean ± sem). (D) Western blot of USP11 expression in HPV+ parental and resistant cells, following 5 μM cisplatin treatment for 24 h compared to vehicle only control. (E) Quantification of three repeats of D (*n* = 5, mean ± sem). (F) USP11 mRNA expression in untreated HPV+ parental and resistant cells (*n* = 3, mean ± sem). (G) Cell viability in HPV− resistant cells in response to cisplatin in presence of USP11 knockdown compared to control (scrambled) siRNA (*n* = 3, mean ± sem). IC50 for control siRNA: 100.6 μM, IC50 for USP11 siRNA: 69.59 μM. (H) Confirmation of USP11 knockdown at protein level in HPV− resistant cells with western blot. (I) Cell viability in HPV+ resistant cells in response to cisplatin in presence of USP11 knockdown compared to control (scrambled) siRNA (*n* = 3, mean ± sem). IC50 for control siRNA: 84.97 μM, IC50 for USP11 siRNA: 88.09 μM, *p* = 0.7476. (J) Confirmation of USP11 knockdown in HPV+ resistant cells at protein level with western blot. (K) Representative images of Gamma H2AX immunofluorescence after USP11 knockdown in HPV− resistant cells compared to control (scrambled) siRNA and 24 h of 50 μM cisplatin treatment compared to vehicle only control. Scale bars, 10 μm. (L) Quantification of mean nuclear intensity in K (*n* = 3, mean ± sem). For quantification of immunofluorescence images, at least 50 cells were quantified per biological repeat. Statistical analysis carried out in B, E, and L using one way-ANOVA with post-hoc Tukey’s test. Statistical analysis carried on C and F using unpaired t test. Statistics carried out in G and I using non-linear regression and extra sum-of-squares F test to compare LogIC50 between datasets. ns = not significant, ∗*p* < 0.05, ∗∗*p* ≤ 0.01, ∗∗∗*p* ≤ 0.001, ∗∗∗∗*p* ≤ 0.0001.

Recently, USP11 has been identified as an R-loop regulator which acts through de-ubiquitination of senataxin, thus reducing its degradation.^44^ Therefore, we wished to investigate whether USP11 may be an appropriate method by which to target senataxin in cisplatin resistance cells, with potential therapeutic implications. In keeping with the lack of change in senataxin expression, there was no significant difference in USP11 expression in HPV− resistant cells (Figures 6A–6C). Upon development of resistance in HPV+ cells there was a significant reduction in USP11 protein expression (Figures 6D–6E), however, there was no observed difference in mRNA level (Figure 6F). The observed downregulation of USP11 in HPV+ resistant cells may be a cellular response to the increased expression of senataxin. In the HPV− resistant cells, depletion of USP11 led to reduced cell viability following cisplatin treatment (Figure 6G), however, depletion of USP11 in HPV+ resistant cells did not affect cell viability in response to cisplatin treatment (Figure 6I). In addition, in HPV− resistant cells, knockdown of USP11 led to increased DNA damage, as measured by γH2AX immunofluorescence (Figures 6K and 6L) and alkaline comet assay, respectively (Figure S3C). Furthermore, in HPV− resistant cells, depletion of USP11 led to an associated reduction in senataxin protein following cisplatin treatment (Figures S4G–S4I).

### In OPSCC tumors which have metastasized to bone, there is evidence of increased R-loops levels by S9.6 immunohistochemistry

**Figure 7:**
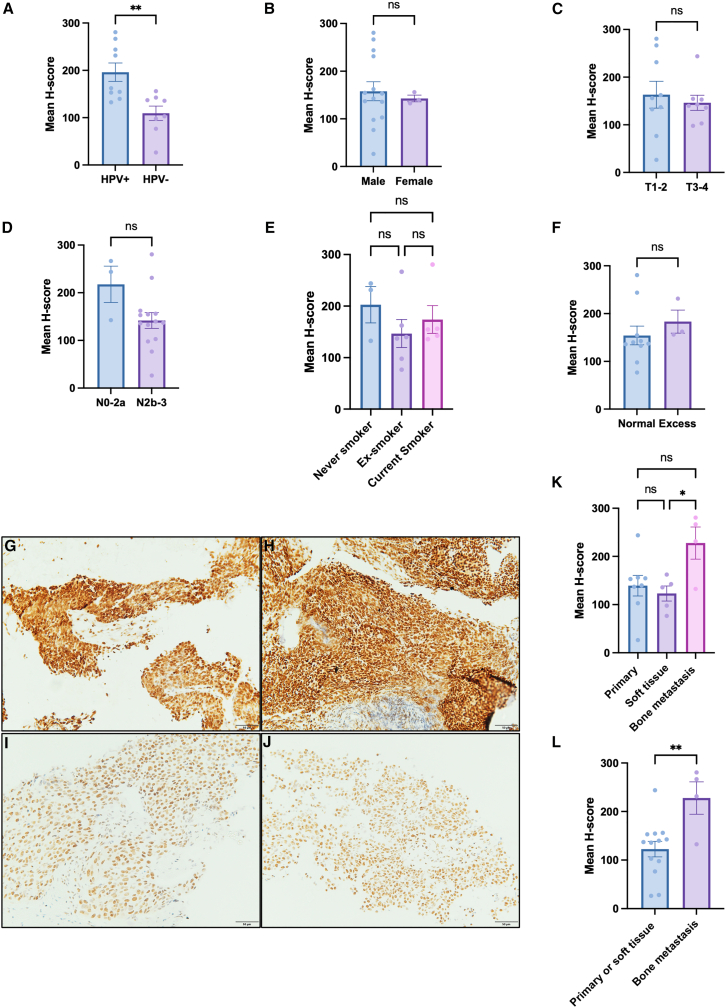
Immunohistochemistry demonstrates higher S9.6 expression in HPV+ tumors and bone metastases (A) Mean H-score by HPV status (*n* = 17, mean ± sem). (B) Mean H-score by gender (*n* = 17, mean ± sem). (C) Mean H-score by tumor stage (*n* = 17, mean ± sem). (D) Mean H-score by nodal stage (*n* = 17, mean ± sem). (E) Mean H-score by smoking status (*n* = 14, mean ± sem). (F) Mean H-score by recorded alcohol consumption (*n* = 14, mean ± sem). Three cases excluded from E and F due to missing clinical data. (G–J) Representative images of primary tumors, soft tissue metastases and bone metastases. (G–H) Representative image of S9.6 staining in bone metastases. (I) Representative image of S9.6 staining in soft tissue (lymph node) metastasis. (J) Representative image of S9.6 staining in primary biopsy (tonsil). (K) Quantification of H-score in primary tumors, soft tissue metastasis and bone metastasis. (L) Quantification of H-score in primary tumors and soft tissue metastasis compared to bone metastasis. Statistical analysis carried out in A–D, F, and L using unpaired t test. Statistics carried out in E and K using one-way ANOVA with post-hoc Tukey’s test. ns = not significant, ∗*p* < 0.05, ∗∗*p* ≤ 0.01.

To explore whether these findings may be replicated *in vivo*, we sought to investigate the R-loop burden using S9.6 immunohistochemistry in a cohort of 17 HPV+ and HPV− tumors which had responded poorly to treatment. All patients were treated non-surgically and the majority received chemoradiotherapy (12/17 patients [71%]). The HPV status of these tumors was confirmed using HPV DNA *in situ* hybridization (ISH) and the specificity of the S9.6 signal was confirmed with RNase H treatment (Figure S5). Following quantification of the S9.6 immunohistochemistry for each of tumors, it was identified that HPV+ tumors have significantly higher S9.6 expression compared to HPV− tumors, however there was no correlation of S9.6 expression with other clinical characteristics including gender, tumor stage, nodal stage, smoking status, or alcohol history (Figures 7A–7F). Within the cohort, there were samples from different sites, including primary tumor, soft tissue metastases, and bone metastases. When the S9.6 expression was compared between these groups, a higher mean S9.6 H-score was observed in the bone metastases when compared to the primary tumors and soft tissue metastases (Figures 7G–7L).

## Discussion

In this paper, we have developed cisplatin resistant HPV+ and HPV− cell lines and uncovered new insights into the biology of platinum resistance in head and neck cancer. We report changes in gene expression upon development of cisplatin resistance in head and neck cancer, including CNGB1 and KRT6A, both of which have been associated with cisplatin resistance in other tumor types.^32^^,^^33^ Furthermore, we identified that pathways including oxidative phosphorylation, keratinization, and WNT signaling were differentially expressed upon development of resistance. Previous studies have investigated alterations in gene expression upon development of cisplatin resistance in HPV− head and neck squamous cell carcinoma, with studies showing cisplatin resistance drives epithelial to mesenchymal transition and a downregulation of keratins,^45^^,^^46^ causes alterations in TNF-α and NF-ΚB signaling^47^ or is associated with oxidative stress.^27^^,^^48^ Although we did not identify alterations in NF-ΚB signaling, we did observe differential expression of oxidative phosphorylation pathways and a downregulation of keratins in line with what has been previously reported.

To examine if R-loops may play a role in the development of cisplatin resistance, we explored R-loop burden in these cells and identified a global increase in R-loops upon development of cisplatin resistance in both HPV− and HPV+ cells. Although one possible explanation is that cellular stress and elevated ROS are leading to increased R-loop levels in resistant cells,^34^^,^^35^^,^^36^ this is unlikely as cisplatin resistance is actually associated with improved recovery from metabolic stress and reduced ROS.^27^^,^^49^ However, there are several other potential explanations as to why R-loops may increase upon development of cisplatin resistance. The first possible explanation is that cells acquire greater genomic instability, with an increase in DNA mutations or structural alterations upon development of cisplatin resistance. Elevated R-loop levels have been shown to be associated with other aggressive tumors, including ETMR^24^ and Ewing sarcoma.^25^ ETMR is a genomically structurally unstable tumor showing overlap of R-loops with regions which have copy number alterations, suggesting R-loops may be a precursor to chromosomal breaks.^24^ Therefore, one possible explanation is cisplatin resistance leads to genomic instability and elevated R-loops may be a cause or consequence of this genomic instability. In support of this theory, two studies analyzed cisplatin resistant ovarian cancer cell lines and their sensitive parental counterparts and identified copy number alterations with gains and loss identified across all chromosomes,^50^^,^^51^ alongside DNA translocations.^51^ Genome wide experiments, such as whole genome sequencing (WGS) and DRIP-seq could be used to investigate this potential explanation further. It is also conceivable that the resistant cells may be utilizing R-loops to directly drive cisplatin resistance by modifying the chromatin landscape and altering gene expression in a favorable manner which aids resistance.^52^^,^^53^ This could be explored further using genome wide R-loop mapping techniques to investigate R-loop occupancy at the promoters of genes known to be involved in cisplatin resistance.

Interestingly, within the RNA-sequencing data, although there were alterations in R-loop regulators upon development of resistance in both HPV+ and HPV− cells, there were fewer differentially expressed regulators in the HPV+ cells despite the greater increase in R-loops. There are a number of potential explanations for this and it is most likely that either post-transcriptional or post-translational changes are causing alterations in R-loop levels through mechanisms that are not detected by standard transcriptome RNA-sequencing.^54^^,^^55^ Alternatively, genomic instability may lead to altered DNA sequences with G-clustering or GC skew which favor R-loop formation but would not lead to an alteration in transcript levels.^20^^,^^21^

Alongside an increase in R-loops globally and at specific loci, HPV+ cells also upregulated senataxin upon development of resistance and depletion of senataxin led to increased sensitivity to cisplatin, increased DNA damage, and increased R-loops both globally and at specific loci. Intriguingly, although HPV− resistant cells did not upregulate senataxin, depletion of senataxin was still able to re-sensitize resistant cells to cisplatin and it is plausible that other R-loop regulators are involved in mediating the changes seen in HPV− cells.^56^ These findings are in agreement with a genome wide CRISPR screen which showed that loss of senataxin sensitized RPE1 cells to cisplatin.^43^ Senataxin has been shown to be important in resolving R-loops at DNA double-strand breaks (DSBs)^57^ and depletion of senataxin reduced cell survival following induced DSBs.^57^ Furthermore, previous studies have demonstrated increased levels of γH2AX and 53BP1 following senataxin knockdown, whereas RAD51 levels were reduced.^57^ Therefore, it could be hypothesized that in the setting of cisplatin resistance, increased levels of senataxin would allow for increased resolution of R-loops formed at DNA DSBs, leading to improved cell viability. However, although the upregulation in senataxin is statistically significant, it is relatively modest; therefore, it is likely that other factors are responsible for the elevated R-loop levels, such as those discussed previously. Nevertheless, senataxin depletion was able to increase sensitivity to cisplatin in an R-loop mediated manner and thus is a potential therapeutic target.

It has recently been demonstrated that high R-loop levels in HPV+ cells are a consequence of E6 repressing p53 transcriptional activity,^58^ however, upon development of cisplatin resistance in HPV+ cells we did not observe a difference in the transcription of E6 (Figure S1C). What contribution viral oncoproteins make to R-loop burden in head and neck cancer remains an unanswered question in the field.

We also investigated whether USP11 depletion was a suitable method of targeting senataxin and thus a potential therapeutic target.^44^ Interestingly, although USP11 was able to re-sensitize HPV− cells to cisplatin in a senataxin-mediated manner (Figures 6 and S4), this was not observed in the HPV+ resistant cells. This can be explained by the observation that HPV+ resistant cells downregulate USP11 protein expression upon cisplatin resistance (Figures 6D–6F), which is likely a cellular response to the elevated expression of senataxin, leading to reduced ubiquitination mediated degradation of senataxin.^44^ Therefore, USP11 may be a potential therapeutic target, but only in cells which do not show a corresponding decrease in USP11 protein expression.

In summary, we have shown that senataxin modulates cisplatin resistance through an R-loop mediated mechanism in HPV+ OPSCC, with potential therapeutic benefits for patients’ who develop cisplatin resistance.

### Limitations of the study

We acknowledge a number of limitations, including the cell line model of cisplatin resistance utilized in this study. A cell line model was chosen as it provided the ability to create cisplatin resistant cell lines and explore the differences between the cisplatin sensitive and resistant cells. It has been shown that lymph node metastasis from OPSCC are heterogeneous in nature,^59^^,^^60^ therefore, evaluation of single clones may not be representative of the complexity of cisplatin resistance *in vivo*. However, the model used in this study allows for manipulation of R-loops that would be impossible in clinical samples. Future work could include evaluating R-loops in tissue samples alongside other markers known to be important in tumor progression (such as immune response and DNA repair), to determine if R-loops are found uniformly throughout a tumor and whether there is any association with other markers which may contribute to treatment resistance. Furthermore, the S9.6 immunohistochemistry was carried out on a relatively small and retrospective cohort and the findings cannot be directly compared with the cell culture data, as for each of the bone metastases there was only these samples to analyze, and the primary tumor was not available for analysis. However, although distant progression is relatively rare in OPSCC, there are a subset of patients who present with distant metastases,^10^^,^^11^^,^^12^^,^^13^^,^^14^ with the second most common site being bone.^14^ Therefore, these findings warrant further investigation to determine if the high level of R-loops in bone metastases may be a potential therapeutic target.

## Resource availability

### Lead contact

Requests for further information should be directed to the lead contact Sherif El-Khamisy (s.el-khamisy@bradford.ac.uk).

### Materials availability

The data supporting this study is available within this manuscript and its supplemental information. This study did not generate any new unique reagents.

### Data and code availability

• The RNA-Sequencing files are available on Gene Expression Omnibus (GEO: GSE279046).
• This study did not generate any unique code.
• Any additional information required to reanalyze the data reported in this paper is available from the lead contact upon request.

## Acknowledgments

This research was funded in whole, or in part, by the 10.13039/100010269Wellcome Trust (Wellcome Trust 4ward North Academy Clinical PhD Fellowship (R120782)). This study was additionally funded by a 10.13039/501100000289Cancer Research UK (CRUK)/Pathological Society Predoctoral Research Bursary (C66701/A27282) and a Pathological Society Equipment Grant (EG20201242). S.E.-K. is supported by a Wellcome Trust Investigator Award (103844), a 10.13039/501100001255Lister Institute of Preventive Medicine Fellowship (137661) and the 10.13039/501100000265Medical Research Council (MR/Y000021/1). For the purpose of Open Access, the author has applied a CC BY public copyright license to any author accepted manuscript version arising from this submission. The authors would like to thank Dr Bernadette Foran for her help in collating the clinical cohort and Hayley Stanhope for her technical assistance.

## Author contributions

Conceptualization: H.C., K.D.H., and S.E.K.; methodology, H.C., I.C., K.D.H., and S.E.K.; validation: H.C.; formal analysis: H.C.; investigation: H.C.; resources: S.E.K. and K.D.H.; data curation: H.C.; writing – original draft: H.C.; writing – review and editing: H.C., I.C., S.E.K., and K.D.H.; supervision: S.E.K. and K.D.H.; project administration: H.C., S.E.K., and K.D.H.; funding acquisition: H.C., K.D.H., I.C., and S.E.K.

## Declaration of interests

The authors declare no competing interests.

## STAR★Methods

### Key resources table

**Table:** 

REAGENT or RESOURCE	SOURCE	IDENTIFIER
**Antibodies**		

Rabbit anti-senataxin antibody	Bethyl laboratories	Cat# A301 – 104A/105A; RRID: AB_873128; RRID: AB_2186221
Rabbit anti-USP11 antibody	Bethyl laboratories	Cat# A301 – 613A; RRID: AB_1211380
Mouse anti-phospho-Histone H2A.X (Ser139) antibody	EMD Millipore	Cat# 05 – 636; RRID: AB_309864
Mouse anti-actin antibody	Sigma	Cat# A5316; RRID: AB_476743
Mouse anti-DNA:RNA hybrid antibody (S9.6)	Isolated from hybridoma by BioServ, Sheffield, UK	N/A
Mouse anti-single stranded DNA antibody	Millipore Sigma	Cat# MAB3868; RRID: AB_570342
Mouse anti-double stranded DNA antibody	Santa Cruz	Cat# sc-58749; RRID: AB_783088

**Biological samples**		

Head and neck tumour samples	Sheffield Teaching Hospitals diagnostic archive	19/YH/0029

**Chemicals, peptides, and recombinant proteins**		

Cis-Diammineplatinum(II) dichloride	Sigma	P4394

**Deposited data**		

fastq files generated from RNA-Sequencing experiment	Gene Expression Omnibus	GEO:GSE279046

**Experimental models: Cell lines**		

HPV-negative head and neck cancer cell line SCC89	University of Pittsburgh	UPCISCC89
HPV-positive head and neck cancer cell line SCC2	University of Pittsburgh	UDSCC2

**Oligonucleotides**		

siRNA sequences – See Table S3	This paper	N/A
RT-qPCR primers – See Table S2	This paper	N/A
DRIP-qPCR primers – See Table S1	This paper	N/A

**Software and algorithms**		

GraphPad Prism	GraphPad Software (Dotmatics)	https://www.graphpad.com/
R	https://www.r-project.org/	Version 4.2.3 "Shortstop Beagle”
ImageJ	Schneider et al.^61^	https://imagej.net/ij/
QuPath	Bankhead et al.^62^	https://qupath.github.io/
Q-Rex	Qiagen	https://www.qiagen.com/us/applications/pcr/thermal-cyclers/q-rex-software
cellSENS imaging software	Olympus	https://evidentscientific.com/en/products/software/cellsens

### Experimental model and study participant details

Two head and neck squamous cell carcinoma cell lines were used in this study, UPCISCC89 (HPV-) and UDSCC2 (HPV+), henceforth referred to as SCC89 and SCC2. These cell lines were received under a material transfer agreement from Professor Gollin, University of Pittsburgh. Both of these cell lines were originally derived from male patients with head and neck cancer, however the age of the patients is unknown.^63^ The presence of high risk HPV16 in the SCC2 cell line was confirmed by testing on a Roche Cobas 6800 instrument and through qPCR for E6 and E7 (Figures S1A–S1D). Both cell lines were STR profiled by NorthGene (Newcastle, UK) and regular mycoplasma testing was undertaken by the core facility service at the School of Clinical Dentistry, University of Sheffield.

Monolayer cell culture was undertaken in a class two biological cabinet and cells were maintained in 37°C incubators with 5% CO_2_. Cells were routinely cultured in low-glucose Dulbecco’s Modified Eagle’s Medium (DMEM), supplemented with 10% fetal bovine serum (FBS), 1% penicillin-streptomycin and 1% L-glutamine.

Ethical approval was sought and obtained to undertake immunohistochemistry on human tissue samples from patients with HPV+ and HPV- head tumours which had responded poorly to treatment (19/YH/0029, n=17). This cohort comprised 3 female and 14 male patients, with an age range at diagnosis of 45 to 87 years (mean age = 59 years). Ethnicity, race and ancestry information was not reported and is therefore not available.

### Method details

#### Generation of cisplatin resistant clones

Cisplatin resistant clones were developed using a dose escalation method as previously described.^64^^,^^65^^,^^66^ Cisplatin was purchased from Sigma-Aldrich (Cis-Diammineplatinum(II) dichloride, P4394) and was dissolved in 0.9% Sodium Chloride (NaCl) to create a stock concentration of 1mM. This was aliquoted and stored at -20°C. SCC89 and SCC2 were treated long-term with cisplatin over a period of 2-3 months, with occasional cisplatin free passages were undertaken to allow cells to recover. IC50 was confirmed with cell viability assays at baseline (Figures 1A/B) and as HPV+ cells had a higher IC50 at baseline (consistent with previous literature^67^), they were treated with a higher dose of cisplatin. SCC89 cells were grown in standard media supplemented with 2.5μM cisplatin, which increased to 5μM cisplatin after 2 passages. SCC2 cells were grown in standard media supplemented with 5μM cisplatin, which increased to 10μM cisplatin after 2 and 4 passages. Cisplatin was added to the media twice weekly. Following dose escalation, cells were grown exclusively in the higher dose of cisplatin. Single cell clones were selected using serial dilution in a 96 well plate format and a single high dose of cisplatin (5μM for SCC89 and 10μM for SCC2). Following selection of single cell clones, cells were grown in cisplatin free media and resistance was confirmed with clonogenic assays. For both the HPV- and HPV+ cells, 3-4 clones were initially screened, and a selected resistant clone was chosen for RNA-sequencing and further experiments (Clone #411 for the HPV- cells and clone #35 for the HPV+ cells (Figures 1C and 1D).

#### Antibodies, siRNA and primers

Details regarding the antibodies, siRNA and primers used in this study can be found in Tables S1–S4. siRNA transfections were undertaken using Lipofectamine RNAiMAX, following the manufacturer’s instructions. USP11 siRNA was used at a final concentration of 20nM and pooled senataxin siRNA was used at a final concentration of 80nM.

#### RNA-sequencing

RNA was extracted using the Monarch Total RNA miniprep kit (New England Biotechnologies (NEB) #T2010) or the Qiagen RNeasy mini kit (74104) according to the manufacturer’s instructions. RNA was quantified using Nanodrop 100 Spectrophotometer (ThermoFisher Scientific) and quality assured using A_260:280_ ratio. cDNA was prepared using the Applied Biosystems High-Capacity cDNA Reverse transcription kit according to the manufacturer’s instructions.

RNA from the parental cells and subsequent resistant clones was sent to Novogene (Cambridge, UK) for mRNA sequencing (polyA library prep, 20 million paired reads per sample, 150bp paired-ended sequencing) on an Illumina NovaSeq. The resulting fastq files were quality assessed using fastQC and combined using multiQC.^68^ The fastq files were aligned and quantified using Salmon^69^ on the high-performance computer (HPC) cluster at the University of Sheffield, using Genome Reference Consortium Human Build 38 (GRCh38). The resulting transcript quantification files were imported into the statistical programme R and differential expression was conducted using DESeq2.^70^ The cut-off for differential expression was set as an adjusted p-value less than 0.05 with a log2fold change of greater than 1. Resulting differentially expressed genes underwent gene ontology analysis using clusterProlifer.^71^

#### Quantitative PCR (RT-qPCR)

cDNA was diluted 1:5-1:20 with nuclease-free water, dependent on the input amount of RNA. Dilutions were kept consistent within the same experiment. Each qPCR reaction contained 5μl of diluted cDNA, 2.8μl of forward and reverse primer at 5μM concentration and 10μl QuantiNova (Qiagen), made up to a final volume of 20μl with nuclease-free water. Standards were run with each reaction on a Rotor-Gene 6000 qPCR machine (Qiagen) and the following thermocycling conditions were used: denaturation at 95°C for 10 minutes, followed by 50 cycles of denaturation at 95° C for 15 seconds, annealing at 5°C below the average melting temperature of the forward and reverse primer for 15 seconds and extension at 72°C for 30 seconds. A melt curve was added to the end of each reaction by increasing the temperature from 72°C to 95°C in 1°C increments at 5 second intervals. CT values were determined for each reaction using the Q-Rex software and analysed using the delta-delta CT method, normalising gene expression to Actin or GAPDH for each reaction.

#### Cell viability assays

MTS assays were undertaken using the CellTiter 96 Aqueous One Solution Cell Proliferation Assay (Promega) or Abcam MTS assay kit (ab197010). Briefly, cells were seeded at an appropriate density in 96 well plates and allowed to attach overnight. Where stated, following 48 hours of transfection and 24 hours of cisplatin treatment, an appropriate amount of MTS reagent was added to each well following the manufacturer’s instructions and the plate incubated at 37°C for 1-4 hours. The plate was subsequently read on using a Tecan spectrophotometer at 490nm.

Clonogenic assays were performed as previously described.^72^ Cells were seeded at a density of 3,000 to 4,000 cells on a 10cm dish and incubated overnight. The following day, cells were treated with the indicated concentrations of cisplatin and left to form colonies for 7-10 days. Following visible colony formation, the plates washed with PBS, fixed with 80% ethanol for 15 minutes, air dried for 5 minutes and stained with 0.5% crystal violet for 30 minutes. The number of colonies on each plate was counted using an automated cell counter (Protos 3) and the surviving fraction was calculated by dividing the number of colonies on treated plates by the number of colonies on untreated plates.

#### Western blotting

Cells were harvested and protein was extracted using lysis buffer (20mM HEPES pH7.4, 2mM MgCl_2_, 40mM NaCl and 1% Triton-X) containing complete mini EDTA protease inhibitor (Roche) at 1:50 ratio, BaseMuncher (Abcam) at 1:1000 ratio and where appropriate PhosSTOP (Sigma) at a final ratio of 1:20. Western blotting was carried out as previously described^44^ in pre-cast 4-15% gels (Invitrogen) or using 4-20% gradient gel.^73^ 30-50μl of lysis buffer was added to the cell pellet and left on ice for 20 minutes with regular vortexing. The samples were centrifuged at 13000 rpm for 15 minutes and the resulting supernatant was stored at -20°C. The protein was quantified with the Bradford Technique, using Coomassie Blue (Thermofisher). Equal amounts of protein were diluted in 5x protein loading buffer and boiled at 95° C for 5 minutes before being loaded onto an SDS-PAGE gel. Samples were run at 120V for 10 minutes, followed by 170V for 1 hour. Gels were transferred onto nitrocellulose membrane using a Trans-Blot Turbo (Bio-Rad), using the pre-set high molecular weight setting. Following transfer, membranes were blocked for 1 hour at room temperature with 5% dried milk in Tris-buffered saline with 0.1% Tween (TBST) and subsequently incubated with primary antibody (Table S4) overnight at 4°C. Following three 5-minute TBST washes, blots were incubated with an appropriate mouse or rabbit HRP secondary antibody (Bio-Rad) at 1:4,000 concentration for 1 hour at room temperature. Three further TBST washes were carried out before visualisation. Blots were visualised using Clarity Western ECL substrate (Bio-Rad), on a ChemiDoc Imaging system (Bio-Rad) and quantified using Image Studio Lite (Licor).

#### Slot blotting

DNA was extracted using a phenol-chloroform method (described below) or with a DNeasy Blood and Tissue Kit (Qiagen), following the manufacturer’s instructions. 400ng or 5μg of DNA was dotted onto a nylon or nitrocellulose membrane respectively using a slot blot apparatus (Hoefer). Where indicated, samples were pre-treated with exogenous RNase H (M0297, NEB), following the manufacturer’s instructions. For the ssDNA antibody the blot was denatured for 10min in 1.5M NaCl, 0.5M NaOH solution and then neutralised for 10min in 0.5M Tris-HCl pH 7.0, 1M NaOH solution. The blots were UV crosslinked (120000uJ/cm^2^), blocked in 5% dried milk powder in TBST for 1 hour at room temperature and subsequently incubated with primary antibody overnight at 4°C (Table S4). Blots were washed three times in TBST for 5 minutes each and incubated with an anti-mouse HRP secondary (Bio-Rad) at 1:2000 dilution for 1 hour at room temperature. Following three 5-minute washes with TBST, blots were visualised and quantified as detailed in the western blotting section.

#### DNA:RNA immunoprecipitation (DRIP-qPCR)

DRIP was undertaken based on method previously published by Sanz and Chédin^39^ and the S9.6 antibody was used for immunoprecipitation which has been shown to selectively bind DNA:RNA hybrids.^74^ DNA was extracted as follows. Cells were harvested and resuspended in 1.6ml TE buffer, pH8 (Thermofisher) with 50μl of 20% SDS and 10 μL of 10mg/mL of proteinase K and incubated overnight at 37°C. The lysate was added to a MaXtract high density tube with an equal amount of phenol/chloroform/isoamyl alcohol and mixed by inversion 5 times. Following centrifugation at 1500g for 10 minutes, the clear supernatant was added to a 15ml tube containing 4ml of 100% ethanol and 160μl of 3M sodium acetate. The DNA was precipitated by mixing on a rotary mixer at 10rpm for 10 minutes and the DNA was spooled out and washed with 80% ethanol three times for 10 minutes each. The DNA was then air dried and resuspended in 125μl of TE buffer. A restriction enzyme digest was then set up to incubate overnight at 37°C containing up to 118.5μl of DNA, 1.5μl of 10m/ml BSA (NEB), 15μl of 2.1 buffer (NEB), 30 units of SSP1 (NEB), 30 units of BSRG1 (NEB), 30 units of ECoR1 (NEB), 30 units of HindIII (NEB), 30 units of XBA1 (NEB), 1.5μl of 100mM spermidine and the reaction was made up to 150μl with nuclease free water. The following day the DNA was added to a phase lock light gel tube (Quantabio), with 100μl of nuclease free water and 250μl of Ultrapure™ phenol:chloroform:isoamyl alcohol (Thermofisher). Following centrifugation at 16000g for 10 minutes, the supernatant was added to 625μl 100% ethanol, 25μl sodium acetate and 1.5μl of glycogen and incubated at -20°C for at least one hour. This was followed by a centrifugation at 16000g for 35 minutes at 4°C. The supernatant was removed and 200μl of 80% ethanol was added followed by a further centrifugation at 16,000g for 10 minutes at 4°C. The supernatant was removed, the DNA pellet was air-dried, resuspended in 50μl of TE buffer, quantified using a Nandrop 100 spectrophotometer and stored at -80°C.

For each condition, 8μg of DNA was diluted in 500μl of TE buffer and 50μl was taken as input. For the RNase H treated sample, an equal amount of DNA (8μg) was treated with 8μl of RNase H in 1x RNase H buffer (NEB) for 5 hours at 37°C. Following RNase H digestion, 400μl of TE buffer was added to the RNase H sample and subsequently treated identically to the non-RNase H treated sample. 52μl of 10x DRIP binding buffer (2.8ml 5M NaCl, 1ml 1M sodium phosphate and 50μl Triton-X in 10ml nuclease free water) and 10μl of S9.6 antibody were added to each sample (RNase H treated and untreated) and incubated for 14-17 hours with 10rpm rotation at 4°C. The following day, 90μl of Protein G beads were washed twice with 700μl 1x DRIP binding buffer for 10 minutes at 10rpm. The DNA was added to the washed beads and incubated for 2 hours at 4°C with 10rpm rotation. The supernatant was discarded, and the beads were washed twice with 750μl of 1X DRIP binding buffer for 15 minutes with 10rpm rotation. 300μl of DRIP elution buffer was then added to the beads with 14μl of 10mg/ml proteinase K and incubated at 55°C for 45 minutes with 10rpm rotation. The resulting supernatant was transferred to a phase lock light gel tube (Quantabio) and phenol-chloroform purification undertaken as detailed in the previous paragraph. The resulting DRIP-DNA was stored at -80°C until qPCR.

DRIP-qPCR was undertaken using QuantiNova SYBR Green-based PCR (Qiagen) on a Rotor-Gene 6000 (Qiagen). Standards were produced by pooling equal amounts of each input and serially diluting 1:10. Each reaction contained 10μl of QuantiNova, 2.8μl of forward and reverse primer pair (at 5μM concentration), 2μl of DNA and 5.2μl of nuclease free water. The cycling conditions are detailed in the quantitative PCR (RT-qPCR) section and the primers are detailed in Table S1.

#### Immunofluorescence

Immunofluorescence was performed as previously described.^44^ Cells were seeded at an appropriate density onto 13mm glass coverslips in a 24 well plate. Following transfection and cisplatin treatment for the time indicated in each figure, the media was removed, and wells washed with 500μl of PBS. Cells were then fixed with 3.7% formaldehyde for 10 minutes at room temperature, followed by permeabilisation with 0.5% Triton-X for 5 minutes. Wells were then washed with 500μl of PBS three times, followed by blocking with 500μl of 3% BSA in PBS-T for 1 hour at room temperature. Wells were incubated with 160μl of primary antibody diluted in 3% BSA for 1 hour at room temperature or overnight at 4°C as follows: Senataxin (1:1000 dilution, Bethyl, A301 – 104A or 105A), USP11 (1:500 dilution, Bethyl, A301 – 613A), γH2AX (1:1000 dilution, EMD Millipore 05 – 636). Following three washes with 500μl of PBS-T the wells were incubated with the following secondary antibodies in the dark for 1 hour at room temperature at 1:500 dilution: Alexa Fluor™ 488 anti-mouse (a11001), Alexa Fluor™ 488 anti-rabbit (a11008) and Alexa Fluor™ 555 anti-rabbit (a21428). Following two washes with 500μl PBS-T and one wash with 500μl PBS the coverslips were removed and allowed to dry for 10 minutes at room temperature before mounting with Immun-Mount™. Visualisation was undertaken on Zeiss Axioplan 2 microscope and images were taken using a 100x objective. The resulting images were analysed in ImageJ using a pre-defined macro to determine the mean nuclear intensity for each cell. Visualisation was undertaken on Zeiss Axioplan 2 microscope and images were taken using a 100x objective. The resulting images were analysed using in ImageJ using a pre-defined macro to determine the mean nuclear intensity for each cell, as previously described.^61^^,^^75^

#### Alkaline single cell gel electrophoresis (comet) assay

Cells were resuspended at 300,000 cells/ml in PBS and mixed with an equal amount of 1.2% low melting point agarose. This was placed underneath a coverslip on top of pre-prepared 0.6% agarose and allowed to set at 4°C for 1 hour. Once set, the coverslips were removed and 1ml of lysis buffer was added to each slide (2.5M NaCl, 10mM Tris HCl, 100mM EDTA pH8.0, 1% Triton X-100, 1% DMSO; pH10) and incubated for 1 hour in the dark at 4°C. Alkaline electrophoresis buffer was prepared by adding 2ml 0.5M EDTA, 5ml 10M NaOH and 10ml DMSO with dH_2_0 to a final volume of 1L. Following lysis, slides were placed in the comet tank with the alkaline electrophoresis buffer for 45 minutes. The comet tank was then run at 12V for 25 minutes and slides were removed and neutralised with 1ml of 0.4M Tris pH7 overnight at 4°C. The slides were visualised on a fluorescence microscope with 1:10,000 SYBR green (Sigma) and quantified using Comet Assay IV. At least 100 cells were scored for each biological replicate, and the mean comet tail was analysed.

#### Immunohistochemistry

Ethical approval was sought and obtained to undertake immunohistochemistry on human tissue samples (19/YH/0029, n=17). Following sectioning, tissue was mounted on SuperFrost Plus™ slides (Epredia) and baked at 60°C for 60 minutes. Slides were dewaxed in xylene twice for 5 minutes each, followed by two 5 minute incubations in 100% ethanol. Endogenous peroxidase activity was blocked with incubation in 3% H_2_0_2_ in methanol for 20 minutes. Slides were briefly washed in PBS, followed by antigen retrieval using sodium citrate buffer in a steamer for 30 minutes (10mM Sodium citrate, 0.05% Tween-20, pH6.0). Where indicated, after antigen retrieval but prior to endogenous blocking, slides were treated with RNase H (25 units RNase H in 1x RNase H buffer) or mock treated with buffer only overnight at 37°C in a humidity chamber filled with dH_2_0. Slides were washed in PBS and a hydrophobic barrier was drawn around the slides with ImmEDGE™ Hydrophobic Barrier Pen (Vector Laboratories). Slides were blocked in 100% horse serum for 1 hour at room temperature in a humidity chamber. The primary antibody was diluted in 100% horse serum (S9.6 at 1:1500 dilution) and slides were incubated overnight at 4°C in a humidity chamber. Following two washes in PBS for 5 minutes each, secondary antibody and ABC complex (VECTSTAIN® Elite® ABC-HRP Kit, Peroxidase (Mouse IgG)) were added according to the manufacturer’s instructions. Following two further washes in PBS for 5 minutes each, staining was visualised using DAB Substrate Kit, Peroxidase (HRP) (Vector Laboratories) for 7 minutes. The slides counterstained with haematoxylin using a Lecia automated stainer, mounted with DPX mountant and visualised on an Olympus light microscope. Images were taken using cellSens image software or slides were scanned using a Leica Aperio CS2 and saved as ∗.svs files. Images and digital slides were analysed using QuPath (Version 0.3.0).^62^

### Quantification and statistical analysis

Statistical analysis was carried out in GraphPad Prism 9 or R (version 4.2.3 "Shortstop Beagle”) using statistical analysis as detailed in each figure legend. In each legend, asterisk indicate the following: ns=not significant, ∗ = p < 0.05, ∗∗ = p ≤ 0.01, ∗∗∗ = p ≤ 0.001, ∗∗∗∗ = p ≤ 0.0001. The data presented are the mean and the standard error of the mean (sem) of three biological replicates, unless otherwise stated in the figure legend. The brightness and contrast has been increased across the entire image in S1G-H, 2I, 6G-J and S5A-D, equally across controls where present.
