# Supplementary material for: Senataxin regulates cisplatin resistance through an R-loop-mediated mechanism in HPV-associated head and neck cancer
Source: iScience. 2025 Aug 13;28(9):113348. doi: 10.1016/j.isci.2025.113348 (PMC12496221; doi:10.1016/j.isci.2025.113348)
Supplement: Document S1. Figures S1–S6 and Tables S1–S4 [file mmc1.pdf]

iScience, Volume *28*

Supplemental information

Senataxin regulates cisplatin resistance through an R-loop-mediated mechanism in HPV-associated head and neck cancer

Hannah Crane, Ian Carr, Keith D. Hunter, and Sherif F. El-Khamisy

Supplementary Figures

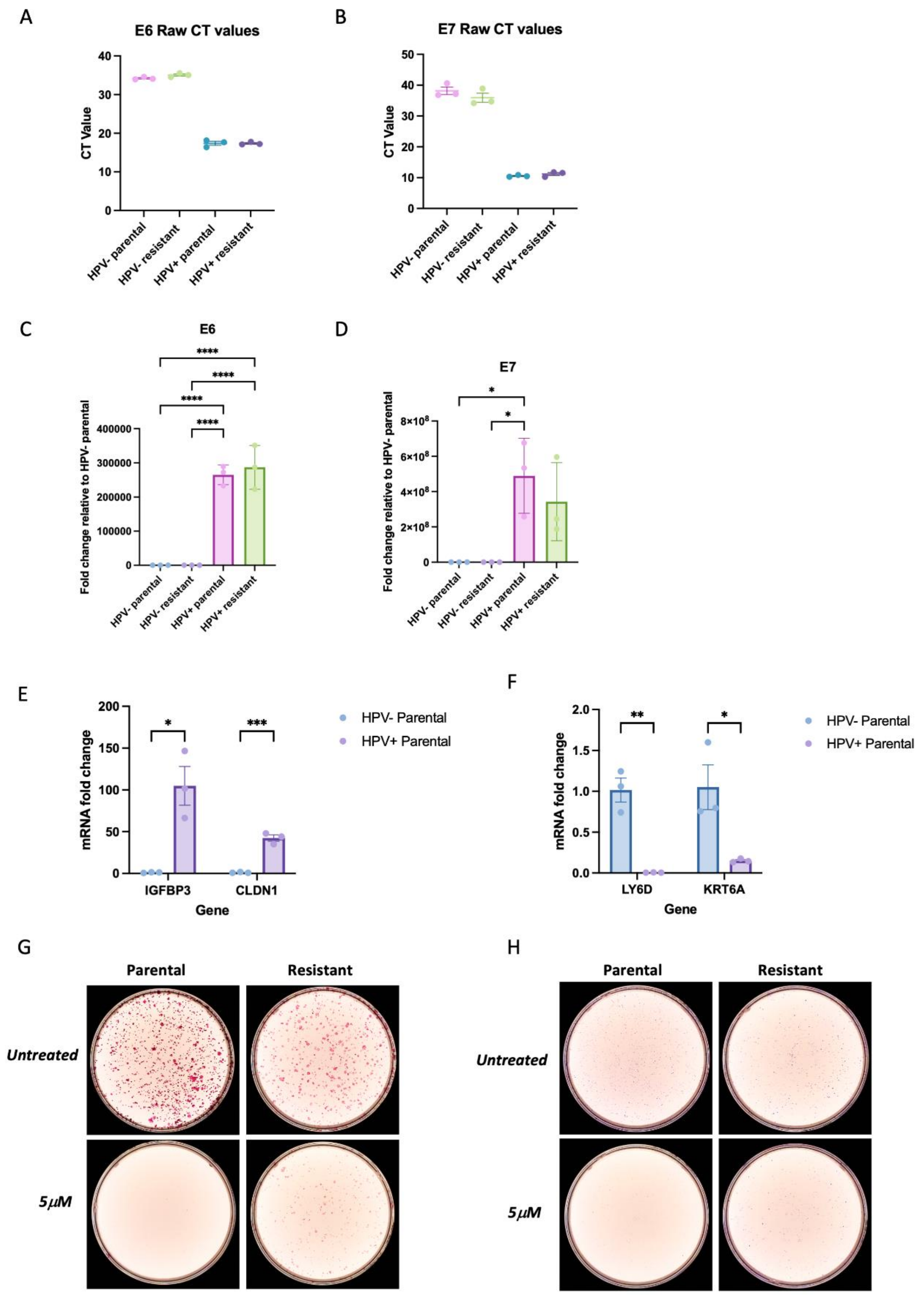

Supplementary Figure 1: qPCR for E6/E7 viral transcripts and validation of RNA-Sequencing analysis confirms the HPV status of the cell lines. A. E6 Raw CT values in HPV+ and HPV- parental and resistant cell lines. B. E7 Raw CT values in HPV+ and HPV- parental and

resistant cell lines. C. Fold change of E6 expression relative to HPV- parental cells (n=3, mean +/- sem). D. Fold change of E7 expression relative to HPV- parental cells (n=3, mean +/- sem). E. qPCR validation of two up-regulated transcripts in the HPV+ parental cells when compared to the HPV- parental cells. F. qPCR validation of two down-regulated transcripts in the HPV+ parental cells when compared to the HPV- parental cells. G. Representative pictures of clonogenic assays with HPV- clone #411, treated with 5μM cisplatin. H. Representative pictures of clonogenic assays with HPV+ clone #35, treated with 5μM cisplatin. Statistical analysis carried out on C and D using one-way ANOVA with Tukey's post-hoc test. Statistical analysis carried out on E and F using multiple unpaired t-tests. ns=not significant, * = $p<0.05$, ** = $p\leq0.01$, *** = $p\leq0.001$, **** = $p\leq0.0001$.

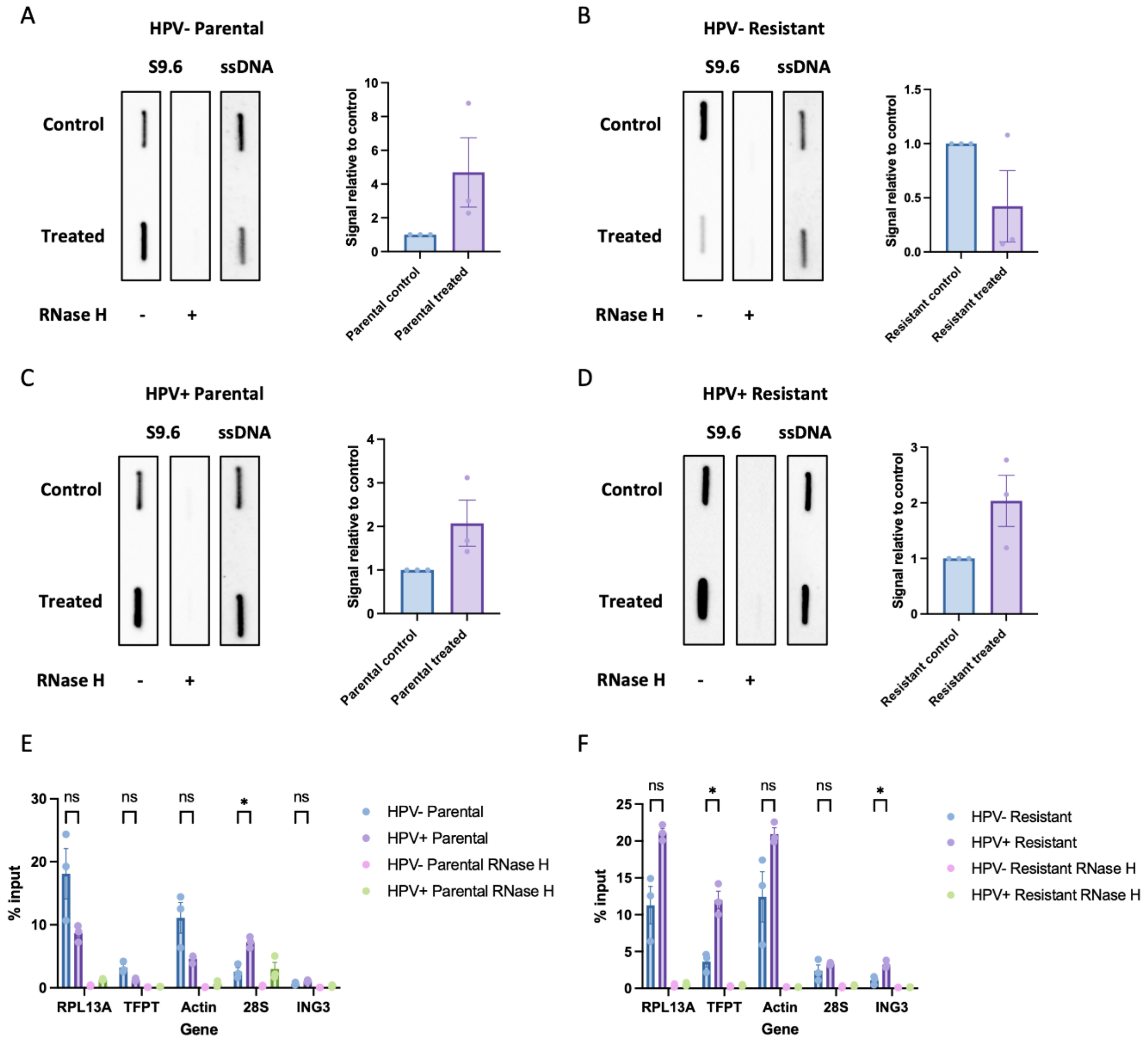

Supplementary Figure 2: Changes in R-loop dynamics at baseline and following cisplatin treatment in HPV- and HPV+ parental and resistant cells. A. S9.6 slot blot showing global R-loop levels following treatment of HPV- parental cells with 5μM cisplatin or vehicle only control for 24 hours (n=3, mean +/- sem). B. S9.6 slot blot showing global R-loop levels following treatment of HPV- resistant cells with 5μM cisplatin or vehicle only control for 24 hours (n=3, mean +/- sem). C. S9.6 slot blot showing global R-loop levels following treatment of HPV+ parental cells with 5μM cisplatin or vehicle only control for 24 hours (n=3, mean +/- sem). D. S9.6 slot blot showing global R-loop levels following treatment of HPV+ resistant cells with 5μM cisplatin or vehicle only control for 24 hours (n=3, mean +/- sem). In A - D, ssDNA (single-stranded DNA) was used as a loading control. E. Percentage input at positive R-loop loci in HPV+ and HPV- parental cells in untreated conditions, with associated RNase H treated controls (n=3, mean +/- sem). F. Percentage input at positive R-

loop loci in HPV+ and HPV- resistant cells in untreated conditions, with associated RNase H treated controls (n=3, mean +/- sem). Statistics carried out in E and F using multiple unpaired t-tests (n=3, mean +/- sem). ns=not significant, * = $p<0.05$.

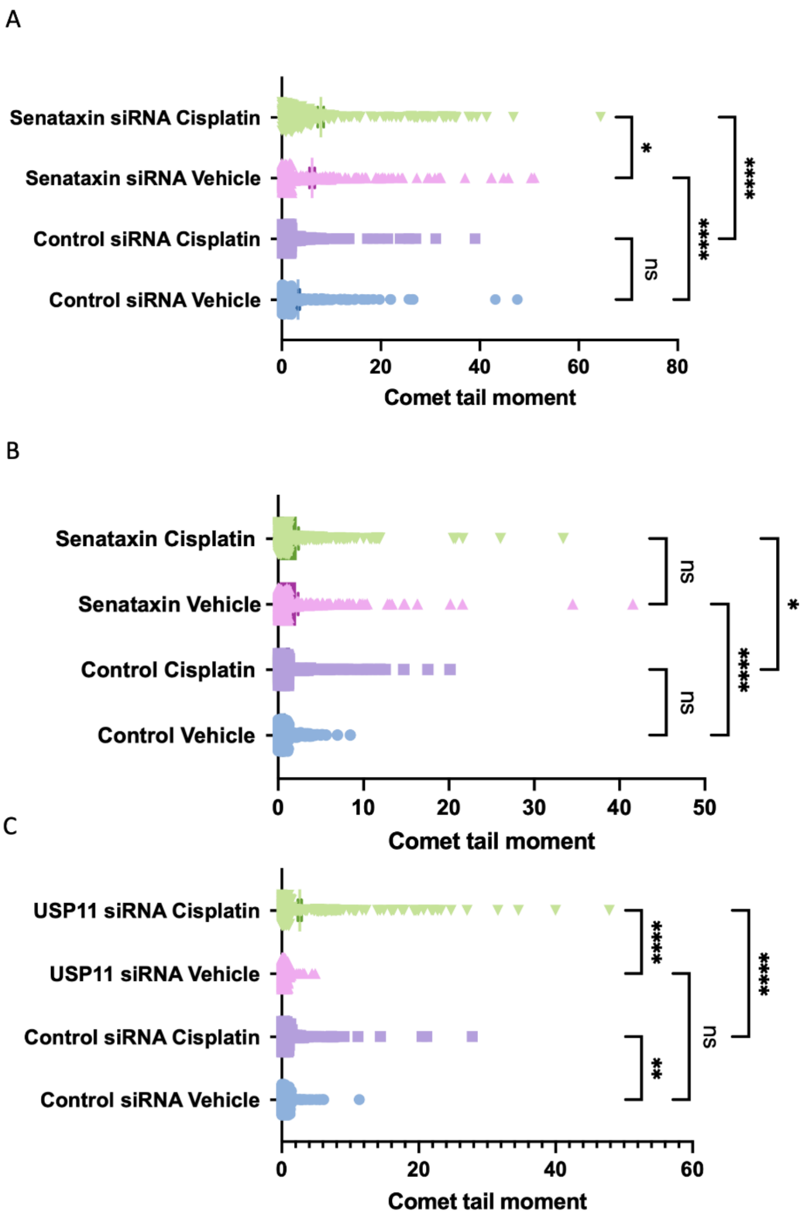

Supplementary Figure 3: Alkaline single gel electrophoresis (comet) assays confirm increased DNA damage following cisplatin treatment in the presence of senataxin or USP11 depletion. A. Comet assay following senataxin depletion and 24 hours of 25μM cisplatin treatment in HPV- resistant cells. B. Comet assay following senataxin depletion and 24 hours of 25μM cisplatin treatment in HPV+ resistant cells. C. Comet assay following USP11 depletion and 24 hours of 50μM cisplatin treatment in HPV- resistant cells. For all figures, mean +/- sem is plotted alongside all data points from 3 biological replicates, with at least 100 cells counted per biological replicate. Statistical analysis carried out with one-way

ANOVA and multiple comparisons carried out using post-hoc Tukey's test. ns=not significant, * = $p<0.05$, ** = $p \leq 0.01$, *** = $p \leq 0.001$, **** = $p \leq 0.0001$.

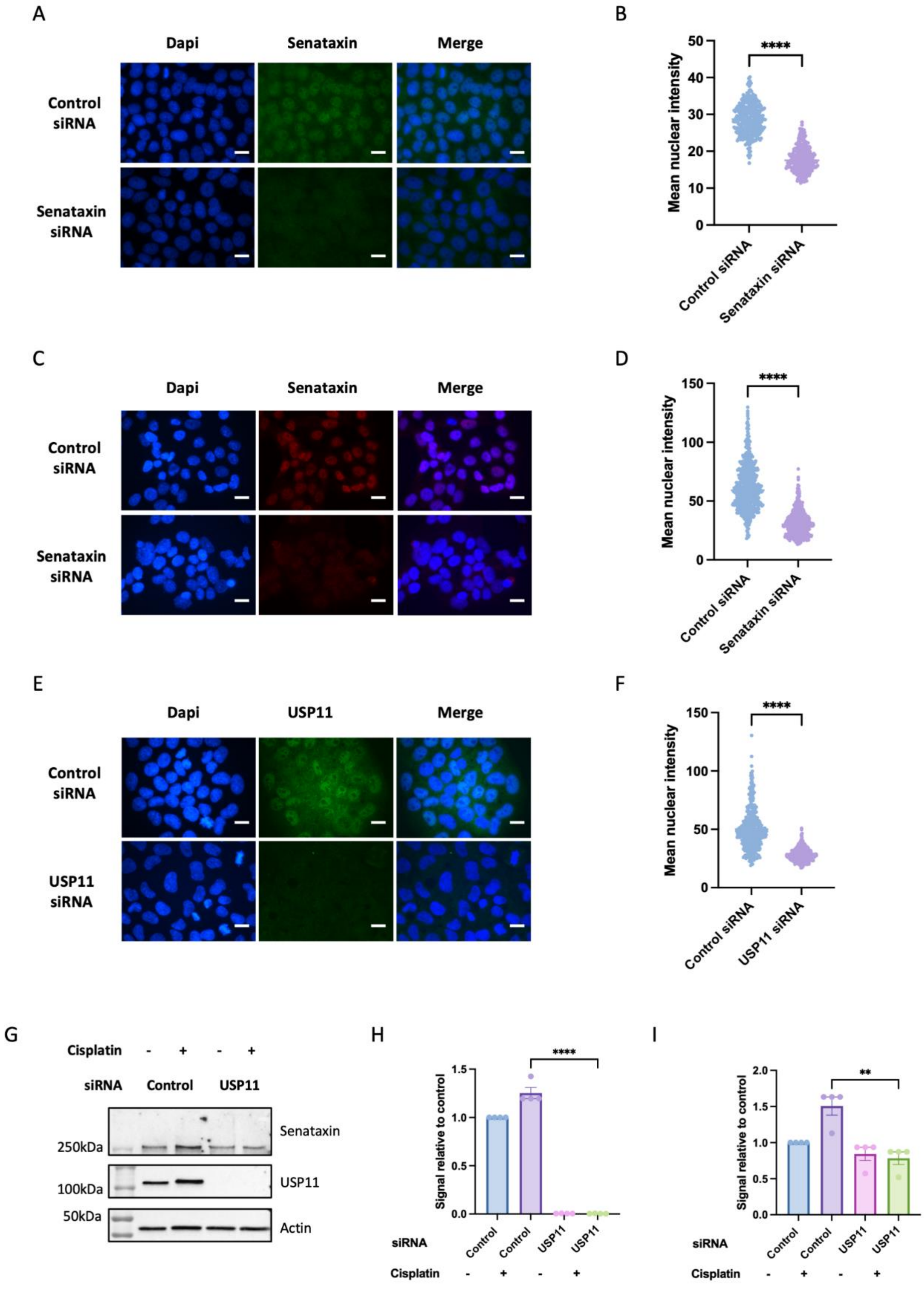

Supplementary Figure 4: Immunofluorescence to confirm Senataxin and USP11 knockdown and western blotting to demonstrate the effect of USP11 depletion on senataxin protein expression. A. Representative images of senataxin immunofluorescence in control (scrambled) and senataxin siRNA treated HPV- resistant cells. B. Quantification of senataxin

nuclear intensity in control and senataxin siRNA groups from Figure A (all data points from two biological replicates plotted, at least 100 cells quantified per biological replicate). C. Representative images of senataxin immunofluorescence in control (scrambled) and senataxin siRNA treated HPV+ resistant cells. D. Quantification of senataxin nuclear intensity in control and senataxin siRNA groups from Figure C (all data points from three biological replicates plotted, at least 100 cells quantified per biological replicate). E. Representative images of USP11 immunofluorescence in control (scrambled) and USP11 siRNA treated HPV- resistant cells. F. Quantification of USP11 nuclear intensity in control and USP11 siRNA groups from Figure E (all data points from three biological replicates plotted, at least 100 cells quantified per biological replicate). G. Representative western blot of HPV- resistant cells treated with USP11 siRNA or control (Scrambled siRNA) and NaCl vehicle or 50μM of cisplatin for 24 hours. H. Quantification of USP11 expression from G (n=4, mean +/- sem). I. Quantification of senataxin expression from G (n=4, mean +/- sem). Statistical analysis carried out in B, D, F, H and I using unpaired t-test, ** = $p \leq 0.01$, **** = $p \leq 0.0001$. Scale bar 10μm.

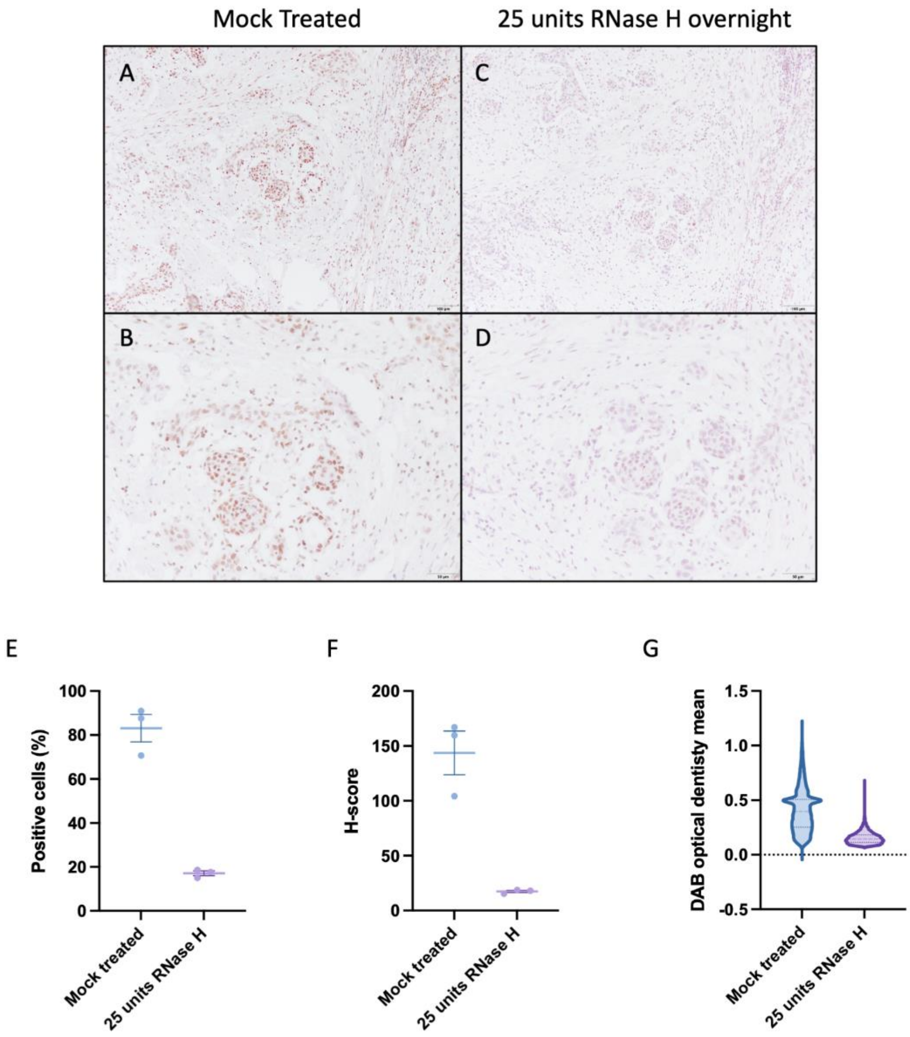

Supplementary Figure 5: RNase H treatment to confirm specificity of S9.6 immunohistochemistry. A-B. Slides were stained with S9.6 antibody at 1:1000 dilution with mock RNase H treatment (buffer only) overnight (B is a higher magnification of image A). C-D. Serial sections of tissue as A-B stained with S9.6 antibody at 1:1000 dilution with 25 units of RNase H treatment (NEB) overnight prior to blocking (C is a higher magnification of image D). E. Percentage of positive cells from three areas of mock treated slide and slide pre-treated with 25 units RNase H. F. H-Score from three areas of mock treated slide and slide pre-treated with 25 units RNase H. G. DAB optical density mean from three areas of mock treated slide and slide pre-treated with 25 units RNase H. Images taken with cellSens software: A and C taken at x100 magnification, B and D taken at x200 magnification.

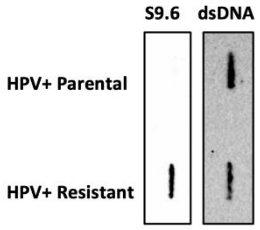

Supplementary Figure 6: Unedited version of S9.6 slot blot in Figure 3I evaluating global differences in R-loops at baseline between HPV+ parental and resistant cells**.**

Supplementary Tables

Name	Forward sequence	Reverse sequence	Source
**RPL13A**	AGGTGCCTTGCTCACAGAGT	GGTTGCATTGCCCTCATTAC	[1]
**TFPT**	TCTGGGAGTCCAAGCAGACT	AAGGAGCCACTGAAGGGTTT	[1]
**FRA3B**	TTAGCCTACTTCAGGGTTTCT	TGGAGAGGTTACTACTGGCA	[2]
**FRA16D**	CAGCCAGCACTCCTTCTCAA	CTCTGTGGAGAAGCCAAGCA	[2]
**Actin 5' Pause**	TTACCCAGAGTGCAGGTGTG	CCCCAATAAGCAGGAACAGA	[3]
**EGR1 promoter**	CATAGGGAAGCCCCTCTTTC	CTTGTGGTGAGGGGTCACTT	[4]
**Actin**	TTTCCGTAGGACTCTCTTCTCT	GTCAGAGAGACAAACACCAGAA	This study (kind gift from Dr Jon Griffin)
**ING3**	TTTTTCTTCTCTAACTACCCTCCCC	GTGCCCTAATCTGAATGACTACA	[5]
**28S**	CAGGGGAATCCGACTGTTTA	ATGACGAGGCATTTGGCTAC	[6]

Supplementary Table 1: DRIP-qPCR primers used in this study and reference where applicable

Name	Forward sequence	Reverse sequence	Source
**E6**	CTGCAATGTTTCAGGACCCA	TCATGTATAGTTGTTTGCAGCTCTGT	[7]
**E7**	ACCGGACAGAGCCCATTACA	GCCCATTAACAGGTCTTCCAAA	[7]
**Actin**	CGCCGCCAGCTCACC	CACGATGGAGGGGAAGACG	This study (Kind gift from Dr Jon Griffin)
**GAPDH**	AGGTCGGAGTCAACGGATTT	ATGAAGGGGTCATTGATGGCA	This study (Kind gift from Dr Jon Griffin)
**USP11**	TGGAAGGCGAGGATTATGTGC	ATGACCTTGCGTTCAATGGGT	[8]
**Senataxin**	CTTCATCCTCGGACATTTGAG	TTAATAATGGCACCACGCTTC	[3]
**CLDN8**	TGAATGTTGCCCAAAAACGTG	GCGATGGGAAGGTATCGAGTATC	[9]
**KRT13**	AGGTGAAGATCCGTGACTGG	GATGACCCGGTTGTTTTCAA	[10]
**LYPD1**	GGCAACTTTTTGCGGATTGTT	CGTTCACCGTGCAATTCACA	[11]
**KRT6A**	AGAGAATGAATTTGTGACTCTGAAGAAG	TACAAGGCTCTCAGGAAGTTGATCT	[12]
**CNGB1**	CACGGCCAGCACAAATA	TTGGGGCTCTCCTCATC	[13]
**CIITA**	CCGACACAGACACCATCAAC	CTTTTCTGCCCAACTTCTGC	[14]
**KRT16**	GACCGGCGGAGATGTGAAC	CTGCTCGTACTGGTCACGC	[15]
**IGFBP3**	AAATGCTAGTGAGTCGGAGGA	CTCTACGGCAGGGACCATATT	[16]
**TGFB1**	TATTGAGCACCTTGGGCACTG	TCTCTGGGCTTGTTTCCTCAC	[17]
**LY6D**	GCTCCCAGACGACATCAGAG	TGTTCGTGGTCTTGCAGAAG	[10]
**CLDN1**	CGAATTTGGTCAGGCTCT	GAAGGTGCAGGTTTTGGA	[18]

Supplementary Table 2: RT-qPCR primers used in this study and reference where applicable

siRNA	siRNA sequence
siControl	5’ UAA UGU AUU GGA ACG GAU 3’
siUSP11	5’ GGA CCG UGA UGA UAU CUU C 3’
siSEXT 1	5’ GCA CGU CAG UCA UGC GUA A 3’
siSEXT 2	5’ GCA AUA AGC UCA UCC UAG U 3’
siSEXT 3	5’ GCU CAA CUC UCC AAA UAG A 3’
siSEXT 4	5’ UAG CAC AGG UUG UUA AUC A 3’

Supplementary Table 3: siRNA sequences

**Antibody**	**Manufacturer**	**Concentration**
Senataxin	Bethyl, A301 – 104A or 105A	Western blot: 1:1000<br>Immunofluorescence: 1:1000
USP11	Bethyl, A301 – 613A	Western blot: 1:1000<br>Immunofluorescence: 1:500
γH2AX	EMD Millipore 05 – 636	Immunofluorescence: 1:1000
Actin	Sigma, A5316	Western blot: 1:1000
S9.6	Isolated from hybridoma by BioServ, Sheffield, UK	Slot blot: 1:500<br>Immunohistochemistry: 1:1500
ssDNA	EMD Millipore, MAB3868	Slot blot: 1:5000
dsDNA	Santa-Cruz, sc-58749	Slot blot: 1:500

Supplementary Table 4: Primary antibodies used in this study.

References

1. Sanz, L. A. & Chédin, F. High-resolution, strand-specific R-loop mapping via S9.6-based DNA-RNA immunoprecipitation and high-throughput sequencing. *Nat. Protoc.* **14**, 1734–1755 (2019).
2. Björkman, A. *et al.* Human RTEL1 associates with Poldip3 to facilitate responses to replication stress and R-loop resolution. *Genes Dev.* **34**, 1065–1074 (2020).
3. Skourti-Stathaki, K., Proudfoot, N. J. & Gromak, N. Human Senataxin Resolves RNA/DNA Hybrids Formed at Transcriptional Pause Sites to Promote Xrn2-Dependent Termination. *Mol. Cell* **42**, 794–805 (2011).
4. Ginno, P. A., Lott, P. L., Christensen, H. C., Korf, I. & Chédin, F. R-Loop Formation Is a Distinctive Characteristic of Unmethylated Human CpG Island Promoters. *Mol. Cell* **45**, 814–825 (2012).
5. Halász, L. *et al.* RNA-DNA hybrid (R-loop) immunoprecipitation mapping: an analytical workflow to evaluate inherent biases. *Genome Res.* **27**, 1063–1073 (2017).
6. Johnston, R. *et al.* The identification of a novel role for BRCA1 in regulating RNA polymerase I transcription. *Oncotarget* **7**, 68097–68110 (2016).
7. Brand, T. M. *et al.* Human papillomavirus regulates HER3 expression in head and neck cancer: Implications for targeted HER3 therapy in HPV+ patients. *Clin. Cancer Res.* **23**, 3072–3083 (2017).
8. Zhou, Z. *et al.* Regulation of XIAP Turnover Reveals a Role for USP11 in Promotion of Tumorigenesis. *EBioMedicine* **15**, 48–61 (2017).
9. Cheng, B., Rong, A., Zhou, Q. & Li, W. CLDN8 promotes colorectal cancer cell proliferation, migration, and invasion by activating MAPK/ERK signaling. *Cancer Manag. Res.* **11**, 3741 (2019).
10. McCray, T., Moline, D., Baumann, B., Griend, D. J. Vander & Nonn, L. Single-cell RNA-Seq analysis identifies a putative epithelial stem cell population in human primary prostate cells in monolayer and organoid culture conditions. *Am. J. Clin. Exp. Urol.* **7**, 123 (2019).
11. Yin, M. *et al.* Comprehensive Analysis of RNA-Seq in Endometriosis Reveals Competing Endogenous RNA Network Composed of circRNA, lncRNA and mRNA. *Front. Genet.* **13**, (2022).
12. Lobo, J. *et al.* Practicability of clinical application of bladder cancer molecular

classification and additional value of epithelial-to-mesenchymal transition: prognostic value of vimentin expression. *J. Transl. Med.* **18**, 303 (2020).

13. Miao, T. W. *et al.* Identification of Survival-Associated Gene Signature in Lung Cancer Coexisting With COPD. *Front. Oncol.* **11**, 600243 (2021).
14. Farina, F. *et al.* Effect of Gliadin Stimulation on HLA-DQ2.5 Gene Expression in Macrophages from Adult Celiac Disease Patients. *Biomedicines* **10**, (2022).
15. Elazezy, M. *et al.* Emerging insights into keratin 16 expression during metastatic progression of breast cancer. *Cancers (Basel).* **13**, 3869 (2021).
16. Ng, E. F. Y., Kaida, A., Nojima, H. & Miura, M. Roles of IGFBP-3 in cell migration and growth in an endophytic tongue squamous cell carcinoma cell line. *Sci. Rep.* **12**, 11503 (2022).
17. Maldonado, L. A. G. *et al.* Influence of tumor cell-derived TGF-β on macrophage phenotype and macrophage-mediated tumor cell invasion. *Int. J. Biochem. Cell Biol.* **153**, (2022).
18. Zhang, Q. *et al.* Screening and validation of lymph node metastasis risk-factor genes in papillary thyroid carcinoma. *Front. Endocrinol. (Lausanne).* **13**, 991906 (2022).
